# Efficacy and safety of dietary polyphenol supplementation in the treatment of non-alcoholic fatty liver disease: A systematic review and meta-analysis

**DOI:** 10.3389/fimmu.2022.949746

**Published:** 2022-09-09

**Authors:** Kailin Yang, Junpeng Chen, Tianqing Zhang, Xiao Yuan, Anqi Ge, Shanshan Wang, Hao Xu, Liuting Zeng, Jinwen Ge

**Affiliations:** ^1^ Key Laboratory of Hunan Province for Integrated Traditional Chinese and Western Medicine on Prevention and Treatment of Cardio-Cerebral Diseases, Hunan University of Chinese Medicine, Changsha, China; ^2^ School of Mechanical Engineering, Hunan University of Science and Technology, Xiangtan, China; ^3^ The First Affiliated Hospital, Department of Cardiovascular Medicine, Hengyang Medical School, University of South China, Hengyang, China; ^4^ The First Hospital of Hunan University of Chinese Medicine, Changsha, China; ^5^ Department of Rheumatology and Clinical Immunology, Peking Union Medical College Hospital, Chinese Academy of Medical Sciences & Peking Union Medical College, Beijing, China; ^6^ Hunan Academy of Chinese Medicine, Changsha, China

**Keywords:** non-alcoholic fatty liver disease, systematic review, meta-analysis, dietary polyphenol, natural plant active ingredients

## Abstract

**Background:**

Dietary polyphenol treatment of non-alcoholic fatty liver disease (NAFLD) is a novel direction, and the existing clinical studies have little effective evidence for its therapeutic effect, and some studies have inconsistent results. The effectiveness of dietary polyphenols in the treatment of NAFLD is still controversial. The aim of this study was to evaluate the therapeutic efficacy of oral dietary polyphenols in patients with NAFLD.

**Methods:**

The literature (both Chinese and English) published before 30 April 2022 in PubMed, Cochrane, Medline, CNKI, and other databases on the treatment of NAFLD with dietary polyphenols was searched. Manual screening, quality assessment, and data extraction of search results were conducted strictly according to the inclusion and exclusion criteria. RevMan 5.3 software was used to perform the meta-analysis.

**Results:**

The RCTs included in this study involved dietary supplementation with eight polyphenols (curcumin, resveratrol, naringenin, anthocyanin, hesperidin, catechin, silymarin, and genistein) and 2,173 participants. This systematic review and meta-analysis found that 1) curcumin may decrease body mass index (BMI), Aspartate aminotransferase (AST), Alanine aminotransferase (ALT), Triglycerides (TG) total cholesterol (TC), and Homeostasis Model Assessment-Insulin Resistance (HOMA-IR) compared to placebo; and curcumin does not increase the occurrence of adverse events. 2) Although the meta-analysis results of all randomized controlled trials (RCTs) did not reveal significant positive changes, individual RCTs showed meaningful results. 3) Naringenin significantly decreased the percentage of NAFLD grade, TG, TC, and low-density lipoprotein cholesterol (LDL-C) and increased high-density lipoprotein cholesterol (HDL-C) but had no significant effect on AST and ALT, and it is a safe supplementation. 4) Only one team presents a protocol about anthocyanin (from *Cornus mas* L. fruit extract) in the treatment of NAFLD. 5) Hesperidin may decrease BMI, AST, ALT, TG, TC, HOMA-IR, and so on. 6) Catechin may decrease BMI, HOMA-IR, and TG level, and it was well tolerated by the patients. 7) Silymarin was effective in improving ALT and AST and reducing hepatic fat accumulation and liver stiffness in NAFLD patients.

**Conclusion:**

Based on current evidence, curcumin can reduce BMI, TG, TC, liver enzymes, and insulin resistance; catechin can reduce BMI, insulin resistance, and TG effectively; silymarin can reduce liver enzymes. For resveratrol, naringenin, anthocyanin, hesperidin, and catechin, more RCTs are needed to further evaluate their efficacy and safety.

## 1 Introduction

Non-alcoholic fatty liver disease (NAFLD) refers to a pathological syndrome characterized by excessive lipid deposition in liver cells caused by alcohol and other definite liver damage factors (mainly including drugs, viral infections, and autoimmunity). It is caused by an imbalance between the input and output of free fatty acid metabolism in the liver (1, 2). Epidemiological surveys have shown that the overweight and obese population is increasing due to huge changes in the global human diet and lifestyle (3, 4). The prevalence of obesity-related chronic diseases such as type 2 diabetes, cardiovascular disease, metabolic syndrome, and NAFLD is also increasing (3, 4). Currently, the global incidence of NAFLD is about 25%, with the highest rates in South America and the Middle East (3, 4). It is particularly noteworthy that the number of NAFLD patients in China has increased dramatically from 18% to nearly 30% in 10 years, and the prevalence rate is more than twice that of developed countries (5). It is estimated that by 2030, the global prevalence of NAFLD patients over 15 years of age will reach 33.5% (6). Therefore, it is of great practical significance to study how to effectively intervene in NAFLD.

NAFLD can be divided into simple fatty liver, non-alcoholic steatohepatitis (NASH), and cirrhosis (7). An early feature of liver cirrhosis is liver fibrosis. At this time, if the patient receives effective treatment, the fibrosis can be alleviated or even cured. However, once fatty liver develops into liver cirrhosis, it will not only increase the risk of liver cancer but also become irreversible for life (8). The results of global epidemiological studies have shown that patients with NAFLD, regardless of whether they have other conditions of metabolic syndrome, are more likely to have heart disease than healthy people, and the probability of dying from myocardial infarction is also higher (9). NAFLD is a highly heterogeneous disease, closely related to genetics, environment, diet, etc. (10). In the natural history of NAFLD, improving NASH can effectively prevent the progression of the disease. The specific pathogenesis of NAFLD/NASH is unclear. The “second hit” theory believes that peripheral adipose tissue dysfunction in insulin resistance leads to lipid breakdown and increases in free fatty acid levels in the blood. Bad living habits also further increase the level of fatty acids in the blood, and excessive fatty acids are transported to the liver, exceeding their transport capacity, and deposited in the liver, resulting in steatosis in the liver (11, 12), which is the “first blow.” Long-term excessive fat deposition induces endoplasmic reticulum stress, mitochondrial dysfunction, and oxidative stress, leading to the release of inflammatory factors, further aggravating liver cell damage and promoting the transformation of simple fatty liver to NASH, which is the “second blow” (13, 14). The peripheral adipose tissue with insulin resistance reduces the secretion of adiponectin and increases the pro-inflammatory factors, which further promote the body’s inflammatory response, aggravate insulin resistance, and form a vicious circle (15, 16). In addition, hepatocyte death, including hepatocyte apoptosis, also plays an important role in the development of NASH. With the progress of research, it is found that the pathogenesis of NAFLD/NASH is extremely complex, and the current view has changed from the “second hit” to the “multiple parallel hit” theory. Various factors such as insulin resistance, adipokines, gut microbiota, genes, and epigenetics may be involved simultaneously (17, 18).

At present, the treatment methods recommended by the latest guidelines at home and abroad include lifestyle intervention, drug treatment, and surgical treatment (19, 20). Polyphenol-rich extracts or isolated polyphenolic monomers from diets have recently received extensive attention for their various biological properties, such as improving metabolism, inhibiting inflammation and oxidative stress, and improving insulin resistance (20, 21). A growing number of randomized controlled trials (RCTs) have shown that dietary polyphenols can improve various pathological indicators in NAFLD patients through multiple pathways (gut, brain, liver, and their interconnected pathways) (22–25). However, the evidence for the treatment of NAFLD with these dietary polyphenols has not been comprehensively evaluated. Therefore, this study conducted a systematic review and meta-analysis of RCTs on the treatment of NAFLD with dietary polyphenols to provide a clinical reference information.

## 2 Materials and methods

### 2.1 Search criteria

#### 2.1.1 Participants

Patients were diagnosed with NAFLD by accepted criteria at the time of publication. There are no restrictions on the patient’s gender, age, ethnicity, and the region where they live and work.

#### 2.1.2 Intervention methods

The experimental group was composed of polyphenol monomers (such as curcumin and resveratrol) or polyphenol-rich plant extracts (such as *C. mas* L. fruit extract) with or without other treatments. The control group was composed of polyphenol-free intervention.

#### 2.1.3 Outcomes

The outcomes were key indicators of NAFLD, including body mass index (BMI), Homeostasis Model Assessment-Insulin Resistance (HOMA-IR), liver enzymes (ALT and AST), blood lipids [total cholesterol (TC), TG, high-density lipoprotein cholesterol (HDL-C), low-density lipoprotein cholesterol (LDL-C)], and adverse events.

#### 2.1.4 Study design

The RCTs of polyphenols for the treatment of NAFLD have no restrictions on language, publication time, etc.

#### 2.1.5 Exclusion criteria

1) Conference abstracts, reviews, and other documents; 2) Duplicate publications of the same research; 3) Documents inconsistent with the research theme; 4) Animal experiments.

### 2.2 Literature retrieval strategy

English databases (including Embase, Medline, PubMed, and Web of Science) and Chinese databases (including CNKI, VIP database, Wanfang Database, Sinomed) were searched to collect the RCTs. The retrieval time was from the establishment of the database to 30 April 2022. Cochrane Library and ClinicalTrials.gov were also searched. The research retrieval strategy of Embase and PubMed was shown in **Table S1**.

### 2.3 Literature screening, extraction, and quality assessment

The process of literature screening, data extraction, and quality assessment was carried out independently by two researchers. The preliminary search was performed in the above databases according to the search strategy, and literature unrelated to dietary polyphenols in the treatment of NAFLD was excluded by reading the title and abstract. Then, the full text of the literature was read, and the literature was further screened according to the search strategy to identify included and excluded literature (26). The risk of bias was independently assessed by two researchers in accordance with The Cochrane Risk Bias Assessment Form provided by the Cochrane Collaboration (27). If there was a disagreement between the two researchers, it would be resolved through consultation with all researchers.

### 2.4 Statistical analysis

Data analysis was performed using RevMan5.3 software provided on the Cochrane website (28). Due to natural differences in the included literature, the I² statistic and H statistic test were used to detect heterogeneity before synthesizing the outcome indicators. When the heterogeneity among the included studies was small (P value >0.1, I² value <50%), the fixed-effects model was selected. When the heterogeneity between studies was large (P value ≤0.1, I² value ≥50%), a random-effects model was used. For continuous variables, mean difference (MD) pooled effect sizes were used, and 95% confidence interval (CI) was used to assess differences in outcome indicators.

## 3 Results

### 3.1 Search results

**Figure 1:**
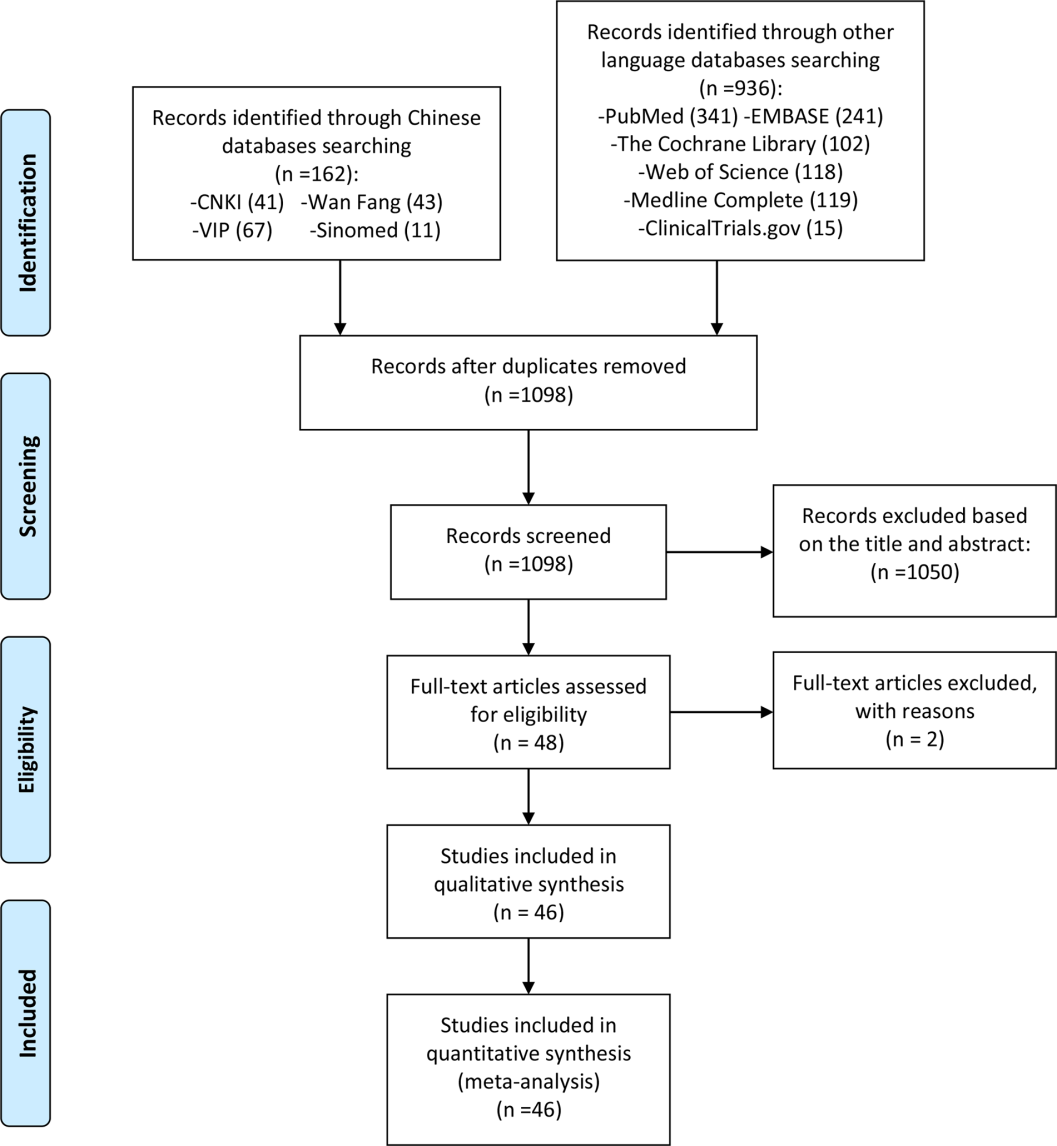
Flow diagram.

After a preliminary search, 1,098 articles were obtained, and 1,050 articles not related to polyphenol supplementation in the treatment of NAFLD were excluded after reading the titles and abstracts. Then, we conducted further screening according to the search criteria and collected a total of 46 records according to the search criteria (29–74), while two records were excluded by reasons (75, 76). The literature screening process is shown in **Figure 1**.

### 3.2 Description of included trials

The RCTs included in this study involved dietary supplementation with eight polyphenols: curcumin, resveratrol, naringenin, anthocyanin, hesperidin, catechin, silymarin, and genistein. Some records are merged together because they belong to the same RCTs: such as Kalhori et al. (29–31), Mirhafez et al. (33–39), Saadati et al. (43, 44), Panahi et al. (47, 48), Farzin et al. (53, 54), Faghihzadeh et al. (55, 56), and Namkhah et al. (58, 59). Among those RCTs, Sangsefidi et al. (60) is a the protocol. Those RCTs came from seven different countries, Iran, Denmark, China, Australia, Pakistan, Italy, and Malaysia, and most of them came from Iran. The study characteristics were shown in **Table 1**.

**Table 1:** The characteristics of the included studies.

Polyphenol	Study	Trial registration number	Country	Sample size		Intervention		Relevant outcomes	Mean age (years)		Duration
				Trial group	Control group	Trial group	Control group		Trial group	Control group	
Curcumin	Kalhori et al. (29–31)	IRCT201406183664N12	Iran	21	21	Turmeric 3,000 mg	Placebo 3,000 mg	BMI, AST, ALT, blood lipid, HOMA-IR	40.38 ± 9.26	42.09 ± 7.23	12 weeks
	Jarhahzadeh et al. (32)	IRCT2015092924262N1	Iran	32	32	Turmeric 2,000 mg	Placebo 2,000 mg	ALT, AST, blood lipid	44.12 ± 8.35	38.56 ± 10.43	12 weeks
	Mirhafez et al. (33–39)	IRCT2015052322381N1	Iran	35	37	Curcumin 250 mg	Placebo 250 mg	BMI, ALT, AST, blood lipid, adverse events	45.0 ± 11.1	43.1 ± 11.6	8 weeks
	Saberi-Karimian et al. (40)	IRCT201702209662N12	Iran	27	28	Curcuminoids 500 mg + piperine 5 mg	Placebo	BMI, ALT, AST, blood lipid	18–70		8 weeks
	Cicero et al. (41)	–	Iran	40	40	Curcumin 200 mg + phosphatidylserine 120 mg + phosphatidylcholine 480 mg + piperine 8 mg	Placebo	ALT, AST, blood lipid, HOMA-IR, adverse events	54 ± 3	53 ± 5	8 weeks
	Moradi Kelardeh et al. (42)	IRCT20190103042219N1	Iran	22	23	Curcumin 80 mg + resistance training or Curcumin 80 mg only	Placebo or resistance training	BMI	Curcumin + resistance training: 64.09 ± 3.33; Curcumin: 66.72 ± 3.03	Placebo: 64.36 ± 2.97; Resistance training: 65.91 ± 3.31	12 weeks
	Saadati et al. (43, 44)	IRCT20100524004010N24	Iran	27	23	Curcumin 1,500 mg	Placebo	ALT, AST, blood lipid, HOMA-IR	46.19 ± 11.5	45.13 ± 10.9	12 weeks
	Panahi et al. (45)	UMIN000033774	Iran	35	35	Curcuminoids 500 mg + piperine 5 mg	Placebo	ALT, AST, blood lipid, adverse events	46.63 ± 2.21	47.51 ± 2.45	12 weeks
	Jazayeri-Tehrani et al. (46)	IRCT2016071915536N3	Iran	42	42	Nanocurcumin 80 mg	Placebo	ALT, AST, blood lipid, HOMA-IR, adverse events	41.8 ± 5.6	42.5 ± 6.2	12 weeks
	Panahi et al. (47, 48)	IRCT2015122525641N2	Iran	44	43	Curcumin 1,000 mg	Placebo	ALT, AST, blood lipid, HOMA-IR, adverse events	44.98 ± 12.59	47.21 ± 10.29	8 weeks
	Rahmani et al. (49)	IRCT2014110511763N18	Iran	37	40	Curcumin formulation 500 mg	Placebo	BMI, ALT, AST, blood lipid, adverse events	46.37 ± 11.57	48.95 ± 9.78	8 weeks
Resveratrol	Heebøll et al. (50)	NCT01464801	Denmark	13	13	Resveratrol 1,500 mg	Placebo	BMI, HOMA-IR, ALT, AST, blood lipid, adverse events	18–70		24 weeks
	Chen et al. (51)	–	China	30	30	Resveratrol 300 mg	Placebo	BMI, HOMA-IR, ALT, AST, blood lipid, adverse events	45.2 ± 10.0	43.5 ± 11.0	12 weeks
	Chachay et al. (52)	–	Australia	10	10	Resveratrol 3,000 mg	Placebo	BMI, HOMA-IR, ALT, AST, blood lipid, adverse events	48.8 ± 12.2	47.5 ± 11.2	8 weeks
	Farzin et al. (53, 54)	IRCT201511233664N16	Iran	25	25	Resveratrol 600 mg	Placebo	BMI, ALT, AST, adverse events	39.78 ± 8.09	38.71 ± 5.76	12 weeks
	Faghihzadeh et al. (55, 56)	NCT02030977	Iran	25	25	Resveratrol 500 mg	Placebo	BMI, ALT, AST, blood lipid, adverse events	44.04 ± 10.10	46.28 ± 9.52	12 weeks
	Kantartzis et al. (57)	NCT01635114	Denmark	53	52	Resveratrol 150 mg	Placebo	ALT, AST, blood lipid, HOMA-IR	18–70		12 weeks
Naringenin	Namkhah et al. (58, 59)	IRCT20131125015536N12	Iran	22	22	Naringenin 200 mg	Placebo	BMI, ALT, AST, blood lipid	44.7 ± 10.7	47 ± 9	4 weeks
Anthocyanin	Sangsefidi et al. (60)	IRCT20180419039359N1	Iran	–	–	*Cornus mas* L. fruit extract	Placebo	Protocol	–	–	12 weeks
Hesperidin	Yari et al. (61)	NCT03734510	Iran	Hesperidin+Flaxseed: 25;Hesperidin: 22	Flaxseed: 22;Placebo: 21	Hesperidin 1,000 mg + Flaxseed 30,000 mg; Hesperidin 1,000 mg	Flaxseed or Placebo	BMI, ALT, AST, blood lipid, HOMA-IR	Hesperidin + Flaxseed: 44.85 ± 10.93; Hesperidin: 45.82 ± 11.69	Flaxseed: 45.04 ± 11.02; Placebo: 46.11 ± 11.63	12 weeks
	Cheraghpour et al. (62)	NCT03377140	Iran	25	24	Hesperidin 1,000 mg	Placebo	ALT, AST, blood lipid, HOMA-IR	47.32 ± 11.66	47.29 ± 13.76	12 weeks
Catechin	Sakata et al. (63)	–	Iran	Low: 5; High: 7	5	Catechin >1,000 mg or 200 mg	Placebo	ALT, AST, blood lipid	Low: 51.4 ± 14.8; High: 47.1 ± 17.2	54.2 ± 8.1	12 weeks
	Tabatabaee et al. (64)	IRCT201404132365N8	Iran	21	24	Catechin 550 mg	Placebo	BMI, ALT, AST, blood lipid	Mean 41	Mean 39.5	12 weeks
	Hussain et al. (65)	–	Pakistan	40	40	Green tea extract 500 mg	Placebo	BMI, ALT, AST, blood lipid, adverse events	25 ± 18	28 ± 15	12 weeks
Silybin	Federico et al. (66)	–	Italy	60	30	Silybin with vitamin D and vitamin E	Placebo	HOMA-IR	54 ± 11	47 ± 10	24 weeks
	Loguercio et al. (67)	–	Italy	69	69	Silybin 94 mg, phosphatidylcholine 194 mg, vitamin E	Placebo	BMI, ALT, AST, BMI	40.8 ± 10.3		48 weeks
	Wah Kheong et al. (68)	NCT02006498	Malaysia	49	50	Silybin 2,100 mg	Placebo	BMI, ALT, AST, blood lipid	49.6 ± 12.7	50.1 ± 10.2	48 weeks
	Hashemi et al. (69)	–	Iran	50	50	Silymarin 280 mg	Placebo	ALT, AST	39.28 ± 11.117	39.0 ± 10.70	24 weeks
	Solhi et al. (70)	IRCT201202159018N1	Iran	33	31	Silymarin 210 mg	Placebo	ALT, AST	43.6 ± 8.3	39.36 ± 10.5	8 weeks
	Anushiravani et al. (71)	IRCT201705016312N4	Iran	30	30	Silymarin 140 mg	Placebo	BMI, blood lipid, ALT, AST, adverse events	47.0 ± 9.1		12 weeks
	Navarro et al. (72)	NCT00680407	Italy	Low: 26 or High: 27	25	Silymarin 420 mg or 700 mg	Placebo	ALT, AST, HOMA-IR, adverse events	Low: 47.3 ± 10.8 or High: 48.2 ± 11.4	49.5 ± 10.9	48 weeks
	Masoodi et al. (73)	–	Iran	50	50	Silymarin 280 mg	Placebo	ALT, AST, BMI, adverse events	48.42 ± 6.75	48.32 ± 5.45	12 weeks
Genistein	Amanat et al. (74)	IRCT201312132480N5	Iran	41	41	Genistein 250 mg	Placebo	BMI, ALT, AST, blood lipid, HOMA-IR, adverse events	44.22 ± 11.80	42.94 ± 9.55	8 weeks

ALT, alanine aminotransferase; AST, aspartate aminotransferase; BMI, body mass index; HOMA-IR, homeostasis model assessment for insulin resistance.

### 3.3 Risk of bias assessment

**Figure 2:**
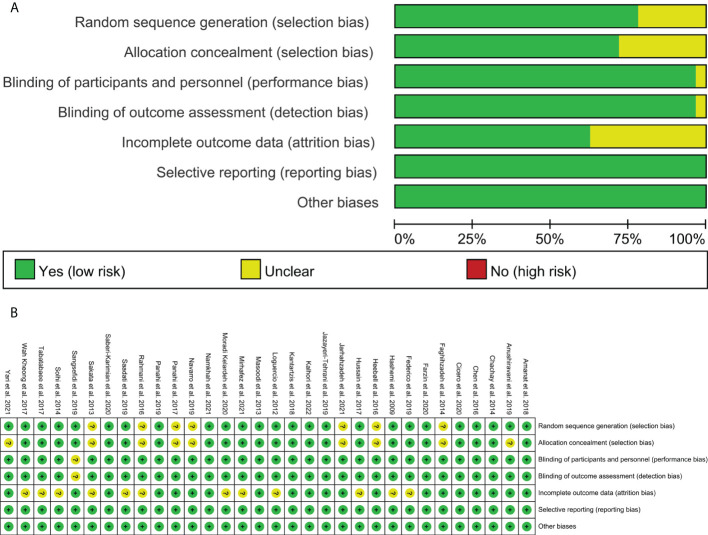
**(A)** Risk of bias graph; **(B)** Risk of bias summary.

The RCTs were assessed by “risk of bias” assessment tools. The summary and graph of risk of bias were shown in **Figure 2**.

### 3.4 Outcomes of curcumin

#### 3.4.1 Body mass index

**Figure 3:**
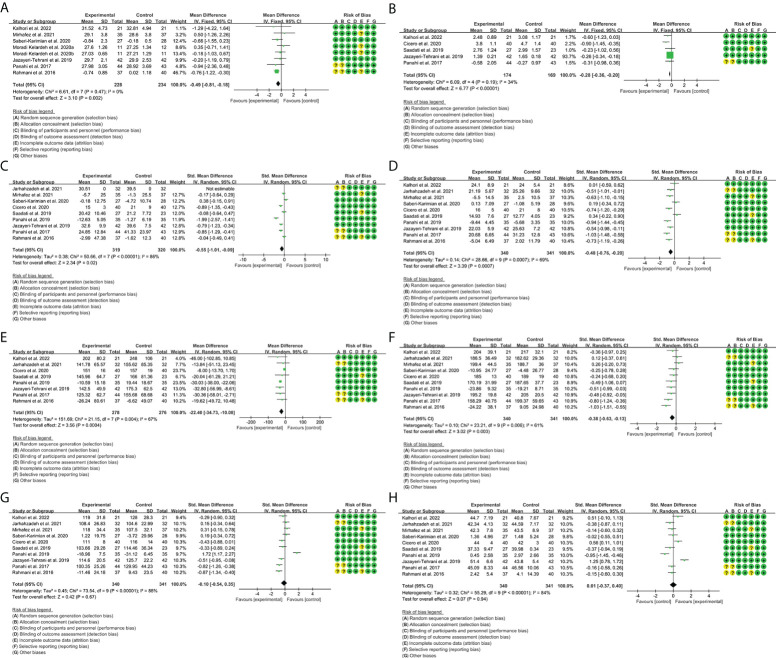
Outcomes in curcumin treatment (**A**: BMI; **B**: HOMA-IR; **C**: ALT; **D**: AST; **E**: TG; **F**: TC; **G**: LDL-C; **H**: HDL-C). CI, confidence interval; SD, standard deviation.

A total of seven RCTs provided evaluable BMI data, involving 228 participants in the experimental group and 234 participants in the control group. The heterogeneity test result was P = 0.47, I^2^ = 0%, suggesting that the heterogeneity among RCTs was low, and the fixed-effects model was used for analysis. The results showed that curcumin reduced BMI compared with placebo [weighted mean difference (WMD) = -0.49, 95% CI (-0.81, -0.18), P = 0.002] (**Figure 3A**). This suggests that curcumin may improve obesity in patients with NAFLD.

#### 3.4.2 Homeostasis model assessment-insulin resistance

A total of five RCTs provided evaluable HOMA-IR data, involving 174 participants in the experimental group and 169 participants in the control group. The heterogeneity test result was P = 0.19, I^2^ = 34%, suggesting that the heterogeneity among RCTs was low, and the fixed-effects model was used for analysis. The results showed that curcumin reduced HOMA-IR compared with placebo [WMD = -0.28, 95% CI (-0.36, -0.20), P < 0.00001] (**Figure 3B**). This suggests that curcumin may improve insulin resistance in patients with NAFLD.

#### 3.4.3 Liver enzymes

A total of 10 RCTs provided evaluable ALT and AST data. For ALT, 319 participants in the experimental group and 320 participants in the control group were involved. The heterogeneity test result was P < 0.00001, I^2^ = 86%, suggesting that the heterogeneity among RCTs was high, and the random-effects model was used for analysis. The results showed that curcumin reduced ALT compared with placebo [SMD = -0.55, 95% CI (-1.01, -0.09), P = 0.02] (**Figure 3C**).

For AST, 340 participants in the experimental group and 341 participants in the control group were involved. The heterogeneity test result was P = 0.0007, I^2^ = 69%, suggesting that the heterogeneity among RCTs was high, and the random-effects model was used for analysis. The results showed that curcumin reduced AST compared with placebo [SMD = -0.48, 95% CI (-0.76, -0.20), P = 0.0007] (**Figure 3D**). These suggest that curcumin may have liver-protective effects.

#### 3.4.4 Blood lipids

A total of eight RCTs provided evaluable TG data, involving 278 participants in the experimental group and 276 participants in the control group. The heterogeneity test result was P = 0.004, I^2^ = 67%, suggesting that the heterogeneity among RCTs was high, and the random-effects model was used for analysis. The results showed that curcumin reduced TG compared with placebo [WMD = -22.40, 95% CI (-34.73, -10.08), P = 0.0004] (**Figure 3E**).

A total of 10 RCTs provided evaluable TC data, involving 340 participants in the experimental group and 341 participants in the control group. The heterogeneity test result was P = 0.006, I^2^ = 61%, suggesting that the heterogeneity among RCTs was high, and the random-effects model was used for analysis. The results showed that curcumin reduced TC compared with placebo [SMD = -0.38, 95% CI (-0.63, -0.13), P = 0.0004] (**Figure 3F**).

A total of 10 RCTs provided evaluable LDL-C data, involving 340 participants in the experimental group and 341 participants in the control group. The heterogeneity test result was P < 0.00001, I^2^ = 88%, suggesting that the heterogeneity among RCTs was high, and the random-effects model was used for analysis. The results showed that there was no significant difference between the curcumin group and the placebo group [SMD = -0.10, 95% CI (-0.54, 0.35), P = 0.67] (**Figure 3G**).

A total of 10 RCTs provided evaluable HDL-C data, involving 340 participants in the experimental group and 341 participants in the control group. The heterogeneity test result was P < 0.00001, I^2^ = 84%, suggesting that the heterogeneity among RCTs was high, and the random-effects model was used for analysis. The results showed that there was no significant difference between the curcumin group and the placebo group [SMD = 0.01, 95% CI (-0.37, 0.40), P = 0.94] (**Figure 3H**). These suggest that curcumin may improve TG and TC level in patients with NAFLD.

#### 3.4.5 Adverse events

Mirhafez et al. (33–39), Cicero et al. (41), Panahi et al. (45), Jazayeri-Tehrani et al. (46), Panahi et al. (47, 48), and Rahmani et al. (49) reported adverse events. They found no significant difference in adverse events in the curcumin group compared with the placebo group (P > 0.05).

### 3.5 Outcomes of resveratrol

#### 3.5.1 Body mass index

**Figure 4:**
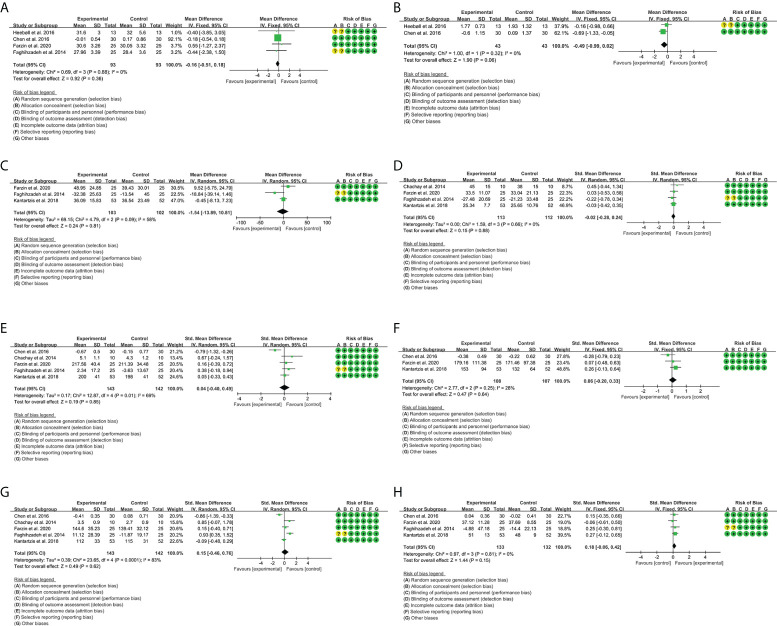
Outcomes in resveratrol treatment (**A**: BMI; **B**: HOMA-IR; **C**: ALT; **D**: AST; **E**: TG; **F**: TC; **G**: LDL-C; **H**: HDL-C).

A total of four RCTs provided evaluable BMI data, involving 93 participants in the experimental group and 93 participants in the control group. The heterogeneity test result was P = 0.88, I^2^ = 0%, suggesting that the heterogeneity among RCTs was low, and the fixed-effects model was used for analysis. The results showed that there was no significant difference between the resveratrol group and the placebo group [WMD = -0.16, 95% CI (-0.51, 0.18), P = 0.36) (**Figure 4A**).

#### 3.5.2 Homeostasis model assessment-insulin resistance

A total of two RCTs provided evaluable HOMA-IR data, involving 43 participants in the experimental group and 43 participants in the control group. The heterogeneity test result was P = 0.32, I^2^ = 0%, suggesting that the heterogeneity among RCTs was low, and the fixed-effects model was used for analysis. The results showed that there was no significant difference between the resveratrol group and the placebo group [WMD = -0.49, 95% CI (-0.99, 0.02), P = 0.06] (**Figure 4B**).

#### 3.5.3 Liver enzymes

A total of three RCTs provided evaluable ALT data, involving 103 participants in the experimental group and 102 participants in the control group. The heterogeneity test result was P = 0.09, I^2^ = 58%, suggesting that the heterogeneity among RCTs was high, and the random-effects model was used for analysis. The results showed that there was no significant difference between the resveratrol group and the placebo group [WMD = -1.54, 95% CI (-13.89, 10.81), P = 0.81] (**Figure 4C**).

A total of four RCTs provided evaluable AST data, involving 113 participants in the experimental group and 112 participants in the control group. The heterogeneity test result was P = 0.66, I^2^ = 0%, suggesting that the heterogeneity among RCTs was high, and the random-effects model was used for analysis. The results showed that there was no significant difference between the resveratrol group and the placebo group [Standard mean difference (SMD) = -0.02, 95% CI (-0.28, 0.24), P = 0.88] (**Figure 4D**).

#### 3.5.4 Blood lipids

A total of three RCTs provided evaluable TG data, involving 108 participants in the experimental group and 107 participants in the control group. The heterogeneity test result was P = 0.25, I^2^ = 28%, suggesting that the heterogeneity among RCTs was low, and the fixed-effects model was used for analysis. The results showed that there was no significant difference between the resveratrol group and the placebo group [SMD = 0.06, 95% CI (-0.20, 0.33), P = 0.64] (**Figure 4E**).

A total of five RCTs provided evaluable TC data, involving 143 participants in the experimental group and 142 participants in the control group. The heterogeneity test result was P = 0.01, I^2^ = 69%, suggesting that the heterogeneity among RCTs was high, and the random-effects model was used for analysis. The results showed that there was no significant difference between the resveratrol group and the placebo group [SMD = 0.04, 95% CI (-0.40, 0.49), P = 0.85] (**Figure 4F**).

A total of five RCTs provided evaluable LDL-C data, involving 143 participants in the experimental group and 142 participants in the control group. The heterogeneity test result was P < 0.00001, I^2^ = 83%, suggesting that the heterogeneity among RCTs was high, and the random-effects model was used for analysis. The results showed that there was no significant difference between the resveratrol group and the placebo group [SMD = 0.15, 95% CI (-0.46, 0.76), P = 0.62] (**Figure 4G**).

A total of four RCTs provided evaluable HDL-C data, involving 133 participants in the experimental group and 132 participants in the control group. The heterogeneity test result was P = 0.81, I^2^ = 0%, suggesting that the heterogeneity among RCTs was low, and the fixed-effects model was used for analysis. The results showed that there was no significant difference between the resveratrol group and the placebo group [SMD = 0.18, 95% CI (-0.06, 0.42), P = 0.15] (**Figure 4H**).

#### 3.5.5 Adverse events

Heebøll et al. (50), Chen et al. (51), Chachay et al. (52), and Farzin et al. (53, 54) reported adverse events. Chen et al. (51) and Farzin et al. (53, 54) did not find obvious adverse events. One case of gastrointestinal side effects and one severe case of febrile leukopenia and thrombocytopenia after 10 days of treatment occurred in the resveratrol group in Heebøll et al. (50). Chachay et al. (52) reported that resveratrol was well tolerated, and the most common adverse event was mild diarrhea.

### 3.6 Outcomes of naringenin

Only Namkhah et al. (58, 59) reported the effects and safety of naringenin in the treatment of NAFLD. They found that naringenin significantly decreased the percentage of NAFLD grade, TG, TC, and LDL-C (P < 0.05) and increased HDL-C (P < 0.05) but had no significant effect on AST and ALT (P > 0.05). Meanwhile, no adverse events were found in their study.

### 3.7 Outcomes of anthocyanin

Only Sangsefidi et al. (60) present a protocol about anthocyanin (from *C. mas* L. fruit extract) in the treatment of NAFLD. It was registered in the Iranian Registry of Clinical Trials (IRCT20180419039359N1). They expect to enroll 80 patients, and the study will last 12 weeks.

### 3.8 Outcomes of hesperidin

Yari et al. (61) and Cheraghpour et al. (62) reported the effects and safety of hesperidin in the treatment of NAFLD. As their indicators could not be pooled for meta-analysis, a general systematic review was performed. Yari et al. (61) found that compared with the control group, hesperidin+flaxseed, hesperidin, and flaxseed could reduce plasma ALT levels and HOMA-IR, fasting blood glucose, and fatty liver index. This suggests that hesperidin and flaxseed supplementation may improve glucose and lipid metabolism while reducing inflammation and hepatic steatosis in NAFLD patients. Cheraghpour et al. (62) found that hesperidin supplementation reduced ALT, γ-glutamyltransferase, TC, TG, hepatic steatosis, high-sensitivity C-reactive protein, Tumor necrosis factor (TNF)-α, Nuclear factor-κB (NF-κB) after 12 weeks of intervention. They speculate that hesperidin plays a role in the management of NAFLD, at least in part, by inhibiting NF-κB activation and improving lipid profiles.

### 3.9 Outcomes of catechin

#### 3.9.1 Body mass index

**Figure 5:**
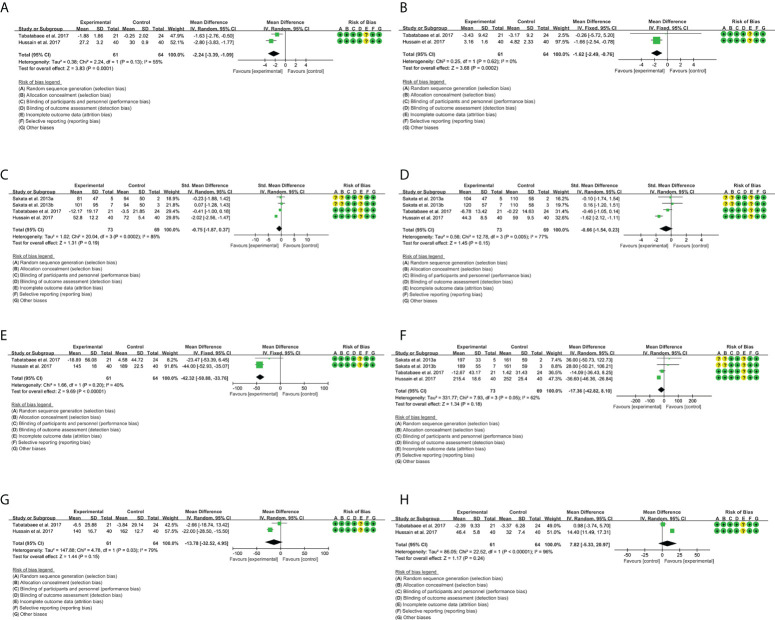
Outcomes in catechin treatment (**A**: BMI; **B**: HOMA-IR; **C**: ALT; **D**: AST; **E**: TG; **F**: TC; **G**: LDL-C; **H**: HDL-C).

A total of two RCTs provided evaluable BMI data, involving 61 participants in the experimental group and 63 participants in the control group. The heterogeneity test result was P = 0.13, I^2^ = 55%, suggesting that the heterogeneity among RCTs was high, and the random-effects model was used for analysis. The results showed that the BMI in the catechin group is lower than that in the placebo group [WMD = -2.24, 95% CI (-3,39, -1.09), P = 0.0001] (**Figure 5A**). This suggests that catechin may improve obesity in patients with NAFLD.

#### 3.9.2 Homeostasis model assessment-insulin resistance

A total of two RCTs provided evaluable HOMA-IR data, involving 61 participants in the experimental group and 63 participants in the control group. The heterogeneity test result was P = 0.62, I^2^ = 0%, suggesting that the heterogeneity among RCTs was low, and the fixed-effects model was used for analysis. The results showed that the HOMA-IR in the catechin group is lower than that in the placebo group [WMD = -1.62, 95% CI (-2.49, -0.76), P = 0.0002] (**Figure 5B**). This suggests that catechin may improve insulin resistance in patients with NAFLD.

#### 3.9.3 Liver enzymes

A total of three RCTs provided evaluable ALT data, involving 73 participants in the experimental group and 69 participants in the control group. The heterogeneity test result was P = 0.0002, I^2^ = 85%, suggesting that the heterogeneity among RCTs was high, and the random-effects model was used for analysis. The results showed that there was no significant difference between the catechin group and the placebo group [SMD = -0.75, 95% CI (-1.87, 0.37), P = 0.19] (**Figure 5C**).

A total of three RCTs provided evaluable AST data, involving 73 participants in the experimental group and 69 participants in the control group. The heterogeneity test result was P = 0.005, I^2^ = 77%, suggesting that the heterogeneity among RCTs was high, and the random-effects model was used for analysis. The results showed that there was no significant difference between the catechin group and the placebo group [SMD = -0.66, 95% CI (-1.54, 0.23), P = 0.15] (**Figure 5D**).

#### 3.9.4 Blood lipids

A total of three RCTs provided evaluable TG data, involving 61 participants in the experimental group and 63 participants in the control group. The heterogeneity test result was P = 0.20, I^2^ = 40%, suggesting that the heterogeneity among RCTs was low, and the fixed-effects model was used for analysis. The results showed that the TG in the catechin group is lower than that in the placebo group [WMD = -42.32, 95% CI (-50.88, -33.76), P < 0.00001] (**Figure 5E**).

A total of three RCTs provided evaluable TC data, involving 73 participants in the experimental group and 69 participants in the control group. The heterogeneity test result was P = 0.01, I^2^ = 69%, suggesting that the heterogeneity among RCTs was high, and the random-effects model was used for analysis. The results showed that there was no significant difference between the catechin group and the placebo group [WMD = -17.36, 95% CI (-42.82, 8.10), P = 0.85] (**Figure 5F**).

A total of two RCTs provided evaluable LDL-C data, involving 61 participants in the experimental group and 64 participants in the control group. The heterogeneity test result was P = 0.03, I^2^ = 79%, suggesting that the heterogeneity among RCTs was high, and the random-effects model was used for analysis. The results showed that there was no significant difference between the catechin group and the placebo group [WMD = -13.78, 95% CI (-32.52, 4.95), P = 0.15] (**Figure 5G**).

A total of two RCTs provided evaluable HDL-C data, involving 61 participants in the experimental group and 64 participants in the control group. The heterogeneity test result was P < 0.00001, I^2^ = 96%, suggesting that the heterogeneity among RCTs was high, and the random-effects model was used for analysis. The results showed that there was no significant difference between the catechin group and the placebo group [WMD = 7.82, 95% CI (-5.33, 20.97), P = 0.24] (**Figure 5H**). These suggest that catechin may improve TG in patients with NAFLD.

#### 3.9.5 Adverse events

Only Hussain et al. (65) reported adverse events. They found that the catechins were well tolerated by the patients, and no major adverse effects were noted during the study period.

### 3.10 Outcomes of silymarin

#### 3.10.1 Body mass index

**Figure 6:**
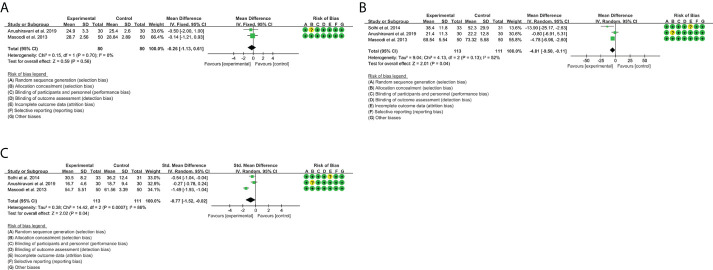
Outcomes in silymarin treatment (**A**: BMI; **B**: ALT; **C**: AST).

A total of four RCTs provided evaluable BMI data, involving 80 participants in the experimental group and 80 participants in the control group. The heterogeneity test result was P = 0.70, I^2^ = 0%, suggesting that the heterogeneity among RCTs was low, and the fixed-effects model was used for analysis. The results showed that there was no significant difference between the silymarin group and the placebo group [WMD = -0.26, 95% CI (-1.13, 0.61), P = 0.56] (**Figure 6A**).

#### 3.10.2 Liver enzymes

A total of three RCTs provided evaluable ALT data, involving 113 participants in the experimental group and 111 participants in the control group. The heterogeneity test result was P = 0.13, I^2^ = 52%, suggesting that the heterogeneity among RCTs was high, and the random-effects model was used for analysis. The results showed that the ALT in the silymarin group was lower than that in the placebo group [WMD = -4.81, 95% CI (-9.50, -0.11), P = 0.04] (**Figure 6B**).

A total of three RCTs provided evaluable AST data, involving 113 participants in the experimental group and 111 participants in the control group. The heterogeneity test result was P = 0.0007, I^2^ = 86%, suggesting that the heterogeneity among RCTs was high, and the random-effects model was used for analysis. The results showed that the ALT in the silymarin group was lower than that in the placebo group [SMD = -0.77, 95% CI (-1.52, -0.02), P = 0.04] (**Figure 6C**). These indicate that silymarin may have liver-protective effects.

#### 3.10.3 Adverse events

Anushiravani et al. (71), Navarro et al. (72), and Masoodi et al. (73) reported adverse events. Anushiravani et al. (71) and Masoodi et al. (73) did not observe adverse events of silymarin during the intervention period. Navarro et al. (72) indicated that no serious adverse events were recorded, and the frequency of side effects was similar and uncommon in the two groups.

### 3.11 Outcomes of genistein

Only Amanat et al. (74) reported outcomes of genistein. They found that serum insulin levels, HOMA-IR, BMI, and TG were reduced after genistein intervention compared with placebo (P < 0.05). However, there were no significant changes in BMI, fasting blood glucose, ALT, and AST between the two groups. No patient reported any serious adverse events as a result. Two minor adverse events were reported by two participants, and one participant in each group had mild gastric distress.

## 4 Discussion

NAFLD has a prevalence of 20%–30% in the general population and >25% in most Asian countries, including China, and is the main cause of abnormal liver enzymes (22). In recent years, the prevalence of metabolic diseases such as obesity, hyperlipidemia, and T2DM has increased year by year, and the prevalence of NAFLD has shown a parallel growth trend. NAFLD is a complex disease regulated by various mechanisms such as glucose and lipid metabolism, genes, environment, and gut microecology (77). Over the past few decades, researchers have been devoted to the exploration of the pathogenesis, prevention, and treatment of NAFLD. There are many differences in the pathogenesis of NAFLD, and the “second hit” hypothesis is currently widely recognized (78). The “second hit” theory holds that the pathogenesis of NAFLD is closely related to insulin resistance; insulin resistance is the central link in the occurrence and development of NAFLD, and abnormal lipid metabolism is the initiating factor (4, 79, 80). Abnormal insulin signaling pathway and lipid metabolism disorder jointly promote the occurrence and development of NAFLD (81). The major sites of P-oxidation of free fatty acids in the liver are mitochondria, microsomes, and peroxisomes. Insulin resistance and hyperinsulinemia promote the release of free fatty acids from peripheral adipose tissue into the liver, accelerate the utilization of free fatty acids by hepatocytes, and promote the synthesis of excess triglycerides in the liver (82). As a result, mitochondrial oxidative phosphorylation and lipid P-oxidation are abnormal, triglyceride transport is abnormal, and very low-density lipoprotein secretion is reduced; it causes benign liver fat accumulation, called “simple fatty liver,” which is the first blow. Steatosis is a necessary condition for the development of NAFLD (83). “One hit” promotes the occurrence and development of the “second hit,” such as activation of inflammatory signaling pathways, mitochondrial dysfunction, and oxidative stress, which contribute to simple fatty liver to steatohepatitis and fatty liver fibrosis (84). Accompanied by the accumulation of visceral fat, the signaling pathway of glucose and lipid metabolism is changed, resulting in the accumulation of fat in the liver, and provides an inflammatory environment and conditions for the occurrence and development of inflammation. This in turn leads to damage to the liver and other tissues and cells, such as oxidative stress, dysregulated protein folding response, lipotoxicity, and apoptosis pathways leading to liver fibrosis, liver cirrhosis, and hepatocellular carcinoma (2, 85, 86). In addition, factors such as endotoxins, hepatotoxic substances, liver tissue overload, and genetic susceptibility produced by intestinal bacterial fermentation also affect processes such as oxidative stress and lipid metabolism through the gut–liver axis (GLA) pathway (87, 88). There is an anatomical and functional connection between the liver and the gut, and 70% of the blood supply to the liver comes from the portal vein. Recent experimental and clinical studies have shown that gastrointestinal microbes can contribute to the occurrence and progression of NAFLD by promoting metabolism and energy acquisition, producing high levels of pro-inflammatory factors, and disrupting local immune cell function through interactions with the host innate immune system (89, 90). Increased intestinal permeability (91), small intestinal bacterial overgrowth (SIBO) (92), and elevated lipopolysaccharide (LPS) (93) may be involved in the pathogenesis of NAFLD. At present, the primary goal for the treatment of NAFLD is to control body weight, improve insulin resistance, and prevent metabolic syndrome and related end-organ lesions. The second is to reduce the deposition of triglycerides in the liver and avoid the “second blow” to form NASH and liver function damage (16, 94).

**Figure 7:**
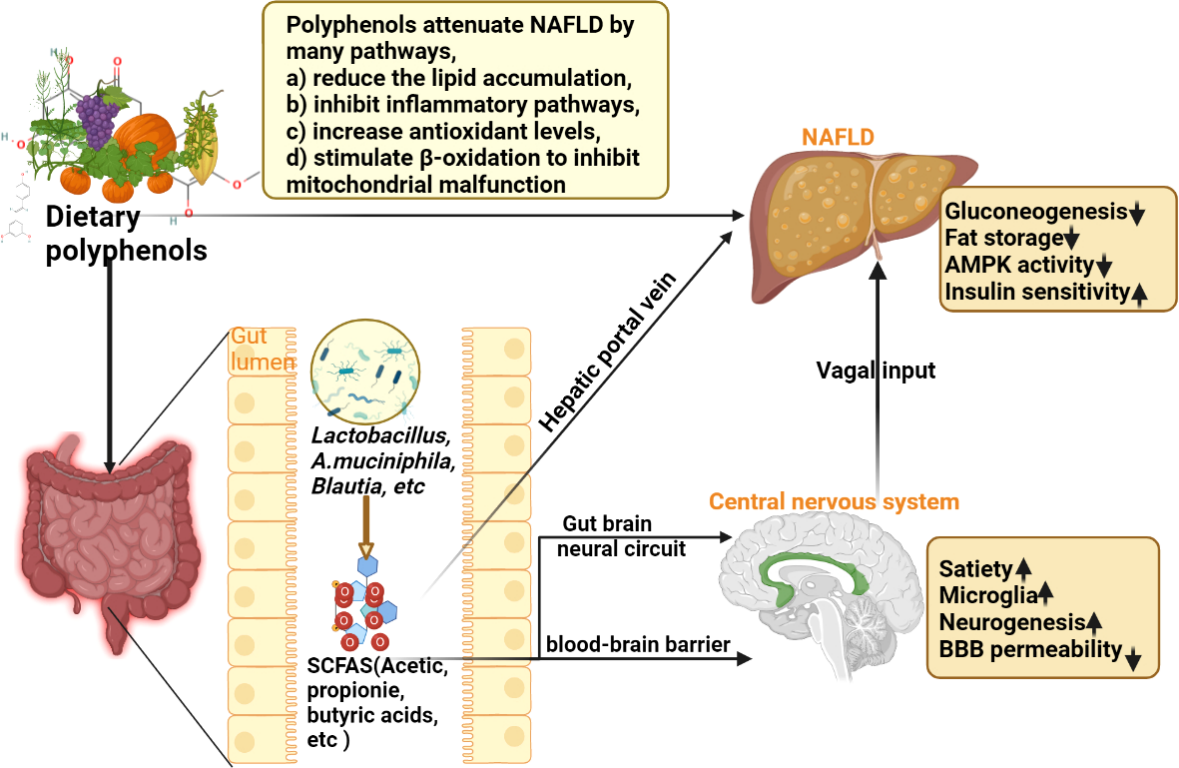
Schematic diagram of the mechanism of dietary polyphenols in the treatment of NAFLD (SCFAS, short-chain fatty acids; NAFLD, non-alcoholic fatty liver disease).

Polyphenols are a diverse class of plant-derived compounds with water-soluble chemical properties (95). They are widely found in fruits, teas, red berries, coffee, red wine, and herbs and are well-known antioxidants and have been proposed as treatments for several metabolic disorders (96). Polyphenols are the most abundant antioxidant compounds in the human diet, and their actions, like those of vitamins, are the cornerstone of the traditionally known beneficial effects of fruits, vegetables, and herbs on a variety of diseases (97, 98). Studies have shown that polyphenols can prevent oxidative stress (99), promote fatty acid β-oxidation, and regulate insulin resistance (99). Furthermore, it has been reported that these compounds may modulate *de novo* lipogenesis by acting on the activity of lipogenic enzymes and improving the expression of lipolytic proteins (100), as well as regulating metabolism by modulating Gut-Liver Axis (GLA) (101). Rafiei et al. (102, 103) observed that several pure polyphenols (such as quercetin, resveratrol, melanin, berberine, catechin, and anthocyanin) effectively protected HepG2 cells from oleic acid-induced steatosis. Some of them protect against mitochondrial dysfunction and aerobic metabolic dysfunction (102, 103). Different polyphenols, such as resveratrol and curcumin, exert their effects through similar molecular targets acting on the Nuclear Factor erythroid 2-Related Factor 2 (Nrf2) pathway, suggesting that these compounds may share the same molecular pathway in lipid metabolism (104, 105). At present, several studies have shown the effect of polyphenols in NAFLD (22, 106) (**Figure 7**). This study summarizes the RCTs of seven polyphenol components in the treatment of NAFLD through a systematic review and meta-analysis and provides the latest clinical evidence for future clinicians and patients. Next, the results of these seven components are described and summarized.

### 4.1 Curcumin

Curcumin is a natural phenolic antioxidant extracted from the rhizomes of ginger plants such as turmeric, curcuma, mustard, curry, and turmeric, and the main chain is unsaturated aliphatic and aromatic groups (107). It has many health-promoting properties that have been shown to include antioxidant, anti-inflammatory, and anticancer effects (108). Recent studies have found that curcumin has made significant progress in the treatment of NAFLD (109). Current studies have found that curcumin and its derivatives can inhibit inflammatory factors in the serum and liver by significantly reducing glucose and lipid metabolism in the serum and liver. It also regulates the metabolic disorder of NAFLD through multiple metabolic pathways [AMPK (Adenosine 5‘-monophosphate-activated protein kinase) signaling pathway and peroxidase proliferator-activated receptor (PPAR) signaling pathway, etc.] (109–111). In addition, curcumin can inhibit hepatic steatosis and hepatic stellate cell activation by activating PPAR-γ (112). The current study also shows that curcumin can directly regulate the gut microbiota to regulate inflammation and energy metabolism in the GLA of NAFLD (113). Studies in rats have shown that approximately 60% of the dose of curcumin suspended in water is absorbed; in addition, suspending it in oil increases the fraction absorbed. About one-third of the oral dose remains in the colon 24 h after oral administration (114). However, unchanged curcumin was not detected in urine or blood nor did it accumulate in tissue or fat. Therefore, it is likely that curcumin undergoes rapid metabolism after absorption by enterocytes or the liver, although other metabolic pathways have not been fully elucidated. The use of 14C-labeled curcumin showed that the major bile metabolites were the glucuronides of tetrahydrocurcumin and hexahydrocurcumin. The minor bile metabolites are dehydroferulic acid and ferulic acid. Only trace amounts of intact curcumin and 6% of total radioactivity are excreted in urine (115). Therefore, if curcumin is fully absorbed, it is mainly excreted through the bile. The effects of all its metabolites are unknown. Luminal curcumin or its derivatives may have local activity on colonic epithelial cells independent of systemic absorption. Although oral curcumin results in therapeutic concentrations in the intestinal mucosa *in vivo* (116), detailed metabolic studies in humans have not been performed. Safety studies have shown that curcumin has no adverse effects reported in rats, guinea pigs, dogs, and monkeys. Curcumin has been shown to be safe in six human trials (117).

This research found that curcumin may decrease BMI, AST, ALT, TG, TC, and HOMA-IR compared to placebo. Meanwhile, curcumin does not increase the occurrence of adverse events. There were six RCTs (29, 32, 42–46) with a follow-up time of 12 weeks and five RCTs (33–41, 47, 49) with a follow-up time of 8 weeks. Kalhori et al. used turmeric 3,000 mg, which is the largest dose in an RCT, while (46) used nanocurcumin 80 mg, and (42) used curcumin 80 mg, which is the smallest dose in an RCT. This suggests that 3 g curcumin continuous intervention for 12 weeks does not increase the occurrence of adverse events. Meanwhile, a phase I human trial using up to 8 g of curcumin per day for 3 months in 25 subjects did not find any toxicity (118). It is not known whether higher doses or longer treatment with curcumin would provide additional benefits. However, because the trial only included adults, this review cannot address the efficacy of curcumin in pediatric patients. Further research is needed to confirm any possible benefit of curcumin treatment in patients of other age groups.

### 4.2 Resveratrol

Resveratrol is a polyphenolic compound that has been shown to be effective in improving liver lesions (119). Resveratrol reverses liver dysfunction associated with nesfatin-1 and glycolipid metabolism in a mouse model of NAFLD, such as transaminases, total bilirubin, TC, LDL-C, blood sugar, insulinemia, and nesfatin-1 (120, 121). In addition, resveratrol improved the histological extent of steatosis and improved behavioral and cognitive impairment caused by NAFLD (21). In terms of glycolipid metabolism, resveratrol can prevent hepatic fat accumulation by enhancing fatty acid β-oxidation and reducing lipogenesis through the AMPK signaling pathway (122). Although the meta-analysis results of all RCTs did not reveal significant positive changes, individual RCTs showed meaningful results. Kantartzis et al. used 75 mg of resveratrol orally twice daily. They found that the resveratrol supplement group had significantly lower levels of liver fat compared to the placebo group, suggesting that resveratrol could prevent liver fat from increasing. Resveratrol was safe and well tolerated during the trial. Chen et al. also found that resveratrol significantly reduced AST, glucose, and LDL-C compared to the placebo group, promoting the role of resveratrol supplementation in the treatment of insulin resistance and its consequences. Faghihzadeh et al. also found that resveratrol reduced ALT and hepatic steatosis. In addition, Farzin et al. (53) investigated the effect of resveratrol on atherogenic risk factors in NAFLD patients. Although resveratrol supplementation reduced BMI and waist circumference compared with the placebo group, no significant changes were found in blood lipids, serum atherosclerotic index, liver enzymes, and blood pressure. Based on current evidence, resveratrol appears to be well tolerated, with few RCTs reporting adverse events. A limitation, however, is that the follow-up periods of these studies were very short (follow-up periods varied from 8 to 24 weeks), and the long-term effects of resveratrol remain unclear. Further evidence is needed to support the efficacy of resveratrol in the management of NAFLD (123).

### 4.3 Naringenin

Naringenin is a natural flavonoid polyphenol with antioxidative stress, anti-inflammatory, improving metabolism, reducing calcium overload, and antiapoptotic effects (124). The current study showed that naringenin ameliorated lipid metabolism disorders in livers by reducing fatty acid uptake and *de novo* lipogenesis and increasing fatty acid oxidation. CD36 and PPAR-α may be specific targets of naringenin (124). Structurally, flavonoid subclasses such as naringenin exert antihyperlipidemic, antidiabetic, and anti-inflammatory functions by attenuating lipid metabolism, glucose metabolism, and inflammation in metabolic syndrome (125). Rodríguez et al. (126) found that naringenin can prevent Streptozotocin (STZ)-induced diabetes-induced liver damage, and it may be a new therapeutic strategy to prevent NAFLD associated with type 1 diabetes. In terms of modulating gut microbiota, naringenin intervention not only reduced body weight gain, hepatic lipid accumulation, and lipogenesis in high-fat diet (HFD)-fed mice but also decreased plasma biochemical parameters. It also altered the gut bacterial community composition, characterized by increased beneficial and decreased harmful bacteria (127). In this systematic review, Namkhah et al. (58, 59) found that naringenin significantly decreased the percentage of NAFLD grade, TG, TC, and LDL-C and increased HDL-C but had no significant effect on AST and ALT. Meanwhile, no adverse events were found in their study.

### 4.4 Anthocyanin

Anthocyanin, a flavonoid polyphenol, is commonly found in various fruits, vegetables, and herbs (128). Due to their excellent antioxidant properties and significant free radical-scavenging ability, anthocyanins have shown definite preventive effects in various stages of the occurrence and development of various cardiovascular diseases and are botanical drugs with broad development prospects (129). As a natural pigment, plant anthocyanins are safe, non-toxic, and rich in resources. They have considerable nutritional and pharmacological effects and have a wide range of impacts on human health. They have great application potential in food, cosmetics, and medicine (130). In terms of safety, anthocyanins as food additives have been proven to have a wide range of safety (131). Evidence shows that anthocyanins in ingested foods are complex flavonoid mixtures, not only non-toxic and non-mutagenic but also these bioflavonoids have various effects on human health maintenance (132, 133). Current studies showed that anthocyanin ameliorated NAFLD by improving lipid and glucose metabolism, increasing antioxidant and anti-inflammatory activities, and modulating gut dysbiosis (134, 135).

### 4.5 Hesperidin

Hesperidin is a flavonoid widely present in citrus fruits, and its chemical structure is dihydroflavonoid glycoside structure (136, 137). It has antioxidation, inhibits inflammation, and improves the molecular mechanism of glucose and lipid metabolism through AMPK/peroxisome proliferator-activated receptor-Gamma coactivator 1 Alpha (PGC-1α) and PPAR signaling pathways (138–140). In addition, studies have shown that HSP can significantly inhibit endoplasmic reticulum stress in oxidative stress in NAFLD models *in vivo* and *in vitro* (141). In this systematic review, Yari et al. found that compared with the control group, hesperidin+flaxseed, hesperidin, and flaxseed could reduce plasma ALT levels and HOMA-IR, fasting blood glucose, and fatty liver index. This suggests that hesperidin and flaxseed supplementation may improve glucose and lipid metabolism while reducing inflammation and hepatic steatosis in NAFLD patients (61). Cheraghpour et al. found that hesperidin supplementation reduced ALT, γ-glutamyltransferase, TC, TG, hepatic steatosis, high-sensitivity C-reactive protein, TNF-α, and NF-κB after 12 weeks of intervention. They speculate that hesperidin plays a role in the management of NAFLD, at least in part, by inhibiting NF-κB activation and improving lipid profiles (62).

### 4.6 Catechin

Catechin is a polyphenolic compound that naturally exists in the dried leaves of *Camellia sinensis* and is the main body of physiologically active substances in green tea extracts. The main structure is 2-phenylbenzopyran (142, 143). Current studies have shown that catechins have hypolipidemic, thermogenic, antioxidative stress, and anti-inflammatory activities, which can reduce the occurrence and progression of NAFLD (144–147). In addition, studies have shown that the effects of green tea on liver enzymes depend on the individual’s health status, and while modest reductions have been observed in NAFLD patients, small increases have been found in healthy subjects (148). This meta-analysis showed that catechin may decrease BMI, HOMA-IR, and TG level. Hussain et al. (65) reported that the catechins were well tolerated by the patients, and no major adverse effects were noted during the study period.

### 4.7 Silymarin

Silymarin, a flavonoid, is the main component of the lipophilic milk thistle extract, which is widely used worldwide as a substance for the treatment of liver disease (149). Studies have shown that silymarin has a good effect on improving high fat-induced fatty liver and insulin resistance and can improve glucose and lipid metabolism and reduce peroxidative damage (150, 151). In patients with biopsy-proven NASH, silymarin improves fibrosis and liver stiffness. For safety, silymarin was found to be safe and well tolerated (68). The current study found that silymarin has anti-inflammatory, immunomodulatory, antifibrotic, antioxidant, and liver regeneration properties in the treatment of NAFLD (152, 153). This meta-analysis and systematic review showed that silymarin was effective in improving ALT and AST and reducing hepatic fat accumulation and liver stiffness in NAFLD patients.

### 4.8 Genistein

Genistein, the main soy isoflavone component of soybean, has been shown to have many biological activities, such as anticancer, antioxidant, and anti-inflammatory effects and inhibition of tyrosine-specific protein kinases (154–157). These properties have made genistein a popular candidate for drug development. The anti-inflammatory activity of isoflavones has been found in several animal studies (158–160). Zhang et al. (125) found that genistein attenuated the pro-inflammatory cytokines TNF-α and IL-6 in metabolic syndrome and improved insulin resistance and fasting blood glucose. Ji et al. (161) found the anti-inflammatory effect of genistein on HFD-induced NASH rats. They found that genistein could improve liver function, slow down NASH progress, and reduce the thiobarbituric acid-reactive substances (TBARS), TNF-α, and IL-6 levels in the serum and liver, such as inhibiting inhibitor of NF-κB α(IκB-α) phosphorylation, nuclear translocation of NF-κB p65 subunit, and activation of c-Jun N-terminal kinase (JNK).

### 4.9 Strength, limitation, and inspiration for the future

The strength of this study is that this systematic review and meta-analysis comprehensively summarizes the current RCTs of dietary polyphenol supplementation in the treatment of NAFLD and evaluates its efficacy and safety, involving dietary supplementation of eight polyphenols (curcumin, resveratrol, naringenin, anthocyanin, hesperidin, catechin, silymarin, and genistein) and 2,173 participants.

The limitations of this study are as follows: 1) There is obvious heterogeneity in some outcomes (such as ALT, AST, TG, TC, LDL-C, HDL-C of curcumin; ALT and AST of resveratrol), and the heterogeneity bias may be due to the selection of population, dietary polyphenol treatment time, dose, selection of dietary polyphenol preparations, and information bias in the data collection process. 2) Although a total of 33 RCTs were included, no more than 10 RCTs were included in each category of dietary polyphenol supplement (naringenin, anthocyanin, and genistein included only one RCT), and the number of participants per RCT was mostly less than 100. 3) The languages of the RCTs included in this study were only Chinese and English, and no RCTs in other languages were found, which may have an impact on the results. 4) The follow-up time of the 33 RCTs included in this study was 8–48 weeks, and there were no observations older than 3 years and earlier than 8 weeks, which may affect the generalization of the results.

Given the above limitations, more research on other classes of polyphenols for the treatment of NAFLD is needed in the future. It is recommended that future RCTs collect treatment data within 8 weeks and beyond 3 years and include larger numbers of participants in order to revise or confirm current conclusions.

## 5 Conclusion

This meta-analysis provides promising findings on the beneficial effects of polyphenol supplementation on NAFLD. These beneficial effects appear to depend on the type of polyphenol: curcumin (80–3,000 mg, 8–12 weeks) can reduce BMI, TG, TC, liver enzymes, and insulin resistance; catechin (500–1,000 mg, 12 weeks) can reduce BMI, insulin resistance, and TG effectively; silymarin (94–2,100 mg, 8–48 weeks) can reduce liver enzymes. These findings provide better insights into the effects of polyphenol supplementation on NAFLD, suggesting that polyphenol supplementation may serve as an inexpensive and long-term NAFLD preventive intervention. However, some polyphenols showed no efficacy (such as resveratrol), and some polyphenols contained fewer RCTs (such as naringenin, anthocyanin, hesperidin, and genistein). Therefore, more RCTs are needed to further evaluate their efficacy and safety.

## Data availability statement

The original contributions presented in the study are included in the article/**Supplementary Material**. Further inquiries can be directed to the corresponding author.

## Authors contributions

KY, JC, TZ, XY, AG, LZ and JG are responsible for the study concept and design. KY, JC, TZ, XY, AG, SW, HX, LZ and JG are responsible for the data collection, data analysis and interpretation; LZ, KY and JC drafted the paper; JG supervised the study; all authors participated in the analysis and interpretation of data and approved the final paper. KY, JC, TZ, XY, AG should be considered joint first author.

## Funding

This work is supported by the National Key Research and Development Project of China (No. 2018YFC1704904), Hunan Provincial Natural Science Foundation of China (2020JJ5424), Hunan University of Chinese Medicine “Double First-Class” Discipline Open Fund Project of Integrated Traditional Chinese and Western Medicine (2020ZXYJH08), Hunan Provincial Department of Education Youth Fund Project (21B30386).

## Conflict of interest

The authors declare that the research was conducted in the absence of any commercial or financial relationships that could be construed as a potential conflict of interest.

## Publisher’s note

All claims expressed in this article are solely those of the authors and do not necessarily represent those of their affiliated organizations, or those of the publisher, the editors and the reviewers. Any product that may be evaluated in this article, or claim that may be made by its manufacturer, is not guaranteed or endorsed by the publisher.

## References

[1] Wong VW . Non-alcoholic fatty liver disease. Lancet (2021) 397(10290):2212–24. doi: 10.1016/S0140-6736(20)32511-3 33894145

[2] Younossi ZM . Non-alcoholic fatty liver disease - a global public health perspective. J Hepatol (2019) 70(3):531–44. doi: 10.1016/j.jhep.2018.10.033 30414863

[3] Huang TD Behary J Zekry A . Non-alcoholic fatty liver disease: A review of epidemiology, risk factors, diagnosis and management. Intern Med J (2020) 50(9):1038–47. doi: 10.1111/imj.14709 31760676

[4] Cotter TG Rinella M . Nonalcoholic fatty liver disease 2020: The state of the disease. Gastroenterology (2020) 158(7):1851–64. doi: 10.1053/j.gastro.2020.01.052 32061595

[5] Arshad T Golabi P Henry L Younossi ZM . Epidemiology of non-alcoholic fatty liver disease in north america. Curr Pharm Des (2020) 26(10):993–7. doi: 10.2174/1381612826666200303114934 32124690

[6] Targher G Tilg H Byrne CD . Non-alcoholic fatty liver disease: A multisystem disease requiring a multidisciplinary and holistic approach. Lancet Gastroenterol Hepatol (2021) 6(7):578–88. doi: 10.1016/S2468-1253(21)00020-0 33961787

[7] Bence KK Birnbaum MJ . Metabolic drivers of non-alcoholic fatty liver disease. Mol Metab (2021) 50:101143. doi: 10.1016/j.molmet.2020.101143 33346069 PMC8324696

[8] Juanola O Martínez-López S Francés R Gómez-hurtado I. . Non-alcoholic fatty liver disease: Metabolic, genetic, epigenetic and environmental risk factors. Int J Environ Res Public Health (2021) 18(10):5227. doi: 10.3390/ijerph18105227 34069012 PMC8155932

[9] Cataldo I Sarcognato S Sacchi D Cacciatore M Baciorri F Mangia A . Pathology of non-alcoholic fatty liver disease. Pathologica (2021) 113(3):194–202. doi: 10.32074/1591-951X-242 34294937 PMC8299321

[10] Forlano R Mullish BH Nathwani R Dhar A Thursz MR Manousou P . Non-alcoholic fatty liver disease and vascular disease. Curr Vasc Pharmacol (2021) 19(3):269–79. doi: 10.2174/1570161118666200318103001 32188385

[11] Stefan N . A global view of the interplay between non-alcoholic fatty liver disease and diabetes. Lancet Diabetes Endocrinol (2022) 10(4):284–96. doi: 10.1016/S2213-8587(22)00003-1 35183303

[12] Muzurović E Mikhailidis DP Mantzoros C . Non-alcoholic fatty liver disease, insulin resistance, metabolic syndrome and their association with vascular risk. Metabolism (2021) 119:154770. doi: 10.1016/j.metabol.2021.154770 33864798

[13] Mahjoubin-Tehran M De Vincentis A Mikhailidis DP Atkin SL Mantzoros CS Jamialahmadi T . Non-alcoholic fatty liver disease and steatohepatitis: State of the art on effective therapeutics based on the gold standard method for diagnosis. Mol Metab (2021) 50:101049. doi: 10.1016/j.molmet.2020.101049 32673798 PMC8324680

[14] Tilg H Adolph TE Dudek M Knolle P . Non-alcoholic fatty liver disease: The interplay between metabolism, microbes and immunity. Nat Metab (2021) 3(12):1596–607. doi: 10.1038/s42255-021-00501-9 34931080

[15] Li H Zhou Y Wang H Zhang M Qiu P Zhang M . Crosstalk between liver macrophages and surrounding cells in nonalcoholic steatohepatitis. Front Immunol (2020) 11:1169. doi: 10.3389/fimmu.2020.01169 32670278 PMC7326822

[16] Abdelmalek MF . Nonalcoholic fatty liver disease: Another leap forward. Nat Rev Gastroenterol Hepatol (2021) 18(2):85–6. doi: 10.1038/s41575-020-00406-0 PMC7791336 33420415

[17] Loomba R Friedman SL Shulman GI . Mechanisms and disease consequences of nonalcoholic fatty liver disease. Cell (2021) 184(10):2537–64. doi: 10.1016/j.cell.2021.04.015 PMC12168897 33989548

[18] Shiha G Korenjak M Eskridge W Casanovas T Velez-Moller P Högström S . Redefining fatty liver disease: An international patient perspective. Lancet Gastroenterol Hepatol (2021) 6(1):73–9. doi: 10.1016/S2468-1253(20)30294-6 33031758

[19] Tacke F Weiskirchen R . Non-alcoholic fatty liver disease (NAFLD)/non-alcoholic steatohepatitis (NASH)-related liver fibrosis: Mechanisms, treatment and prevention. Ann Transl Med (2021) 9(8):729. doi: 10.21037/atm-20-4354 33987427 PMC8106094

[20] Głuszyńska P Lemancewicz D Dzięcioł JB Razak Hady H . Non-alcoholic fatty liver disease (nafld) and bariatric/metabolic surgery as its treatment option: A review. J Clin Med (2021) 10(24):5721. doi: 10.3390/jcm10245721 34945016 PMC8706342

[21] Abenavoli L Larussa T Corea A Procopio AC Boccuto L Dallio M . Dietary polyphenols and non-alcoholic fatty liver disease. Nutrients (2021) 13(2):494. doi: 10.3390/nu13020494 33546130 PMC7913263

[22] Simental-Mendía LE Gamboa-Gómez CI Guerrero-Romero F Simental-Mendía M Sánchez-García A Rodríguez-Ramírez M . Beneficial effects of plant-derived natural products on non-alcoholic fatty liver disease. Adv Exp Med Biol (2021) 1308:257–72. doi: 10.1007/978-3-030-64872-5_18 33861449

[23] Salomone F Godos J Zelber-Sagi S . Natural antioxidants for non-alcoholic fatty liver disease: molecular targets and clinical perspectives. Liver Int (2016) 36(1):5–20. doi: 10.1111/liv.12975 26436447

[24] Bagherniya M Nobili V Blesso CN Sahebkar A . Medicinal plants and bioactive natural compounds in the treatment of non-alcoholic fatty liver disease: A clinical review. Pharmacol Res (2018) 130:213–40. doi: 10.1016/j.phrs.2017.12.020 29287685

[25] Ji Y Yin Y Sun L Zhang W . The molecular and mechanistic insights based on gut-liver axis: Nutritional target for non-alcoholic fatty liver disease (nafld) improvement. Int J Mol Sci (2020) 21(9):3066. doi: 10.3390/ijms21093066 PMC7247681 32357561

[26] Deeks JJ Higgins JP Altman DG . Chapter 16: Special topics in statistics. In: Higgins JP Green S , editors. Cochrane handbook for systematic reviews of interventions. (UK: The Cochrane Collaboration) (2020) 2020.

[27] Deeks JJ Higgins JP Altman DG . Chapter 8: Assessing risk of bias in included studies. In: Higgins JP Green S , editor. Cochrane handbook or systematic reviews of interventions version 6.1.0. (UK: The Cochrane Collaboration) (2020). 2020

[28] Deeks JJ Higgins JP Altman DG . Chapter 9: Analyzing data and undertaking meta-analyses. In: Higgins JP Green S , editors. Cochrane handbook for systematic reviews of interventions. (UK: The Cochrane Collaboration) (2020). 2020

[29] Kalhori A Rafraf M Navekar R Ghaffari A Jafarabadi MA . Effect of turmeric supplementation on blood pressure and serum levels of sirtuin 1 and adiponectin in patients with nonalcoholic fatty liver disease: A double-blind, randomized, placebo-controlled trial. PrevNutr Food Sci (2022) 27(1):37–44. doi: 10.3746/pnf.2022.27.1.37 PMC9007706 35465117

[30] Ghaffari A Rafraf M Navekar R Sepehri B Asghari-Jafarabadi M Ghavami SM . Turmeric and chicory seed have beneficial effects on obesity markers and lipid profile in non-alcoholic fatty liver disease (NAFLD). Int J VitamNutr Res (2019) 89(5-6):293–302. doi: 10.1024/0300-9831/a000568 31017556

[31] Navekar R Rafraf M Ghaffari A Asghari-Jafarabadi M Khoshbaten M . Turmeric supplementation improves serum glucose indices and leptin levels in patients with nonalcoholic fatty liver diseases. J Am Coll Nutr (2017) 36(4):261–7. doi: 10.1080/07315724.2016.1267597 28443702

[32] Jarhahzadeh M Alavinejad P Farsi F Husain D Rezazadeh A . The effect of turmeric on lipid profile, malondialdehyde, liver echogenicity and enzymes among patients with nonalcoholic fatty liver disease: A randomized double blind clinical trial. DiabetolMetabSyndr (2021) 13(1):112. doi: 10.1186/s13098-021-00731-7 PMC8524923 34663438

[33] Mirhafez SR Rezai A Dehabeh M Nobakht M Gh BF Bidkhori M Sahebkar A . Efficacy of phytosomal curcumin among patients with non-alcoholic fatty liver disease. Int J VitamNutr Res (2021) 91(3-4):278–86. doi: 10.1024/0300-9831/a000629 31818232

[34] Mirhafez SR Azimi-Nezhad M Dehabeh M Hariri M Naderan RD Movahedi A . The effect of curcumin phytosome on the treatment of patients with non-alcoholic fatty liver disease: A double-blind, randomized, placebo-controlled trial. Adv Exp Med Biol (2021) 1308:25–35. doi: 10.1007/978-3-030-64872-5_3 33861434

[35] Hariri M Gholami A Mirhafez SR Bidkhori M Sahebkar A . A pilot study of the effect of curcumin on epigenetic changes and DNA damage among patients with non-alcoholic fatty liver disease: A randomized, double-blind, placebo-controlled, clinical trial. Complement Ther Med (2020) 51:102447. doi: 10.1016/j.ctim.2020.102447 32507446

[36] Mirhafez SR Farimani AR Dehhabe M Bidkhori M Hariri M Ghouchani BF . Effect of phytosomal curcumin on circulating levels of adiponectin and leptin in patients with non-alcoholic fatty liver disease: A randomized, double-blind, placebo-controlled clinical trial. J Gastrointestin Liver Dis (2019) 28:183–9. doi: 10.15403/jgld-179 31204416

[37] Chashmniam S Mirhafez SR Dehabeh M Hariri M Azimi Nezhad M Nobakht M GhBF . A pilot study of the effect of phospholipid curcumin on serum metabolomic profile in patients with non-alcoholic fatty liver disease: A randomized, double-blind, placebo-controlled trial. Eur J Clin Nutr (2019) 73(9):1224–35. doi: 10.1038/s41430-018-0386-5 30647436

[38] Mirhafez SR Dehabeh M Hariri M Farimani AR Movahedi A Naderan RD . Curcumin and piperine combination for the treatment of patients with non-alcoholic fatty liver disease: A double-blind randomized placebo-controlled trial. Adv Exp Med Biol (2021) 1328:11–9. doi: 10.1007/978-3-030-73234-9_2 34981468

[39] Mirhafez SR Farimani AR Gholami A Hooshmand E Tavallaie S Nobakht M Gh BF . The effect of curcumin with piperine supplementation on pro-oxidant and antioxidant balance in patients with non-alcoholic fatty liver disease: A randomized, double-blind, placebo-controlled trial. Drug Metab Pers Ther (2019) 34(2):1–7. doi: 10.1515/dmpt-2018-0040 31145689

[40] Saberi-Karimian M Keshvari M Ghayour-Mobarhan M Salehizadeh L Rahmani S Behnam B . Effects of curcuminoids on inflammatory status in patients with non-alcoholic fatty liver disease: A randomized controlled trial. Complement Ther Med (2020) 49:102322. doi: 10.1016/j.ctim.2020.102322 32147075

[41] Cicero AFG Sahebkar A Fogacci F Bove M Giovannini M Borghi C . Effects of phytosomal curcumin on anthropometric parameters, insulin resistance, cortisolemia and non-alcoholic fatty liver disease indices: A double-blind, placebo-controlled clinical trial. Eur J Nutr (2020) 59(2):477–83. doi: 10.1007/s00394-019-01916-7 PMC7058573 30796508

[42] Moradi Kelardeh B Rahmati-Ahmadabad S Farzanegi P Helalizadeh M Azarbayjani MA . Effects of non-linear resistance training and curcumin supplementation on the liver biochemical markers levels and structure in older women with non-alcoholic fatty liver disease. J Bodyw Mov Ther (2020) 24(3):154–60. doi: 10.1016/j.jbmt.2020.02.021 32825982

[43] Saadati S Hatami B Yari Z Shahrbaf MA Eghtesad S Mansour A . The effects of curcumin supplementation on liver enzymes, lipid profile, glucose homeostasis, and hepatic steatosis and fibrosis in patients with non-alcoholic fatty liver disease. Eur J Clin Nutr (2019) 73(3):441–9. doi: 10.1038/s41430-018-0382-9 30610213

[44] Saadati S Sadeghi A Mansour A Yari Z Poustchi H Hedayati M . Curcumin and inflammation in non-alcoholic fatty liver disease: A randomized, placebo controlled clinical trial. BMC Gastroenterol (2019) 19(1):133. doi: 10.1186/s12876-019-1055-4 31345163 PMC6659284

[45] Panahi Y Valizadegan G Ahamdi N Ganjali S Majeed M Sahebkar A . Curcuminoids plus piperine improve nonalcoholic fatty liver disease: A clinical trial. J Cell Biochem (2019) 120(9):15989–96. doi: 10.1002/jcb.28877 31168845

[46] Jazayeri-Tehrani SA Rezayat SM Mansouri S Qorbani M Alavian SM Daneshi-Maskooni M . Nano-curcumin improves glucose indices, lipids, inflammation, and nesfatin in overweight and obese patients with non-alcoholic fatty liver disease (NAFLD): A double-blind randomized placebo-controlled clinical trial. Nutr Metab (Lond) (2019) 16:8. doi: 10.1186/s12986-019-0331-1 30705687 PMC6348610

[47] Panahi Y Kianpour P Mohtashami R Jafari R Simental-Mendía LE Sahebkar A . Efficacy and safety of phytosomal curcumin in non-alcoholic fatty liver disease: A randomized controlled trial. Drug Res (Stuttg) (2017) 67(4):244–51. doi: 10.1055/s-0043-100019 28158893

[48] Panahi Y Kianpour P Mohtashami R Jafari R Simental-Mendía LE Sahebkar A . Curcumin lowers serum lipids and uric acid in subjects with nonalcoholic fatty liver disease: A randomized controlled trial. J Cardiovasc Pharmacol (2016) 68(3):223–9. doi: 10.1097/FJC.0000000000000406 27124606

[49] Rahmani S Asgary S Askari G Keshvari M Hatamipour M Feizi A . Treatment of non-alcoholic fatty liver disease with curcumin: A randomized placebo-controlled trial. Phytother Res (2016) 30(9):1540–8. doi: 10.1002/ptr.5659 27270872

[50] Heebøll S Kreuzfeldt M Hamilton-Dutoit S Kjær Poulsen M Stødkilde-Jørgensen H Møller HJ . Placebo-controlled, randomised clinical trial: High-dose resveratrol treatment for non-alcoholic fatty liver disease. Scand J Gastroenterol (2016) 51(4):456–64. doi: 10.3109/00365521.2015.1107620 26784973

[51] Chen S Zhao X Ran L Wan J Wang X Qin Y . Resveratrol improves insulin resistance, glucose and lipid metabolism in patients with non-alcoholic fatty liver disease: A randomized controlled trial. Dig Liver Dis (2015) 47(3):226–32. doi: 10.1016/j.dld.2014.11.015 25577300

[52] Chachay VS Macdonald GA Martin JH Whitehead JP O’Moore-Sullivan TM Lee P . Resveratrol does not benefit patients with nonalcoholic fatty liver disease. Clin Gastroenterol Hepatol (2014) 12(12):2092–103.e1-6. doi: 10.1016/j.cgh.2014.02.024 24582567

[53] Farzin L Asghari S Rafraf M Asghari-Jafarabadi M Shirmohammadi M . No beneficial effects of resveratrol supplementation on atherogenic risk factors in patients with nonalcoholic fatty liver disease. Int J VitamNutr Res (2020) 90(3-4):279–89. doi: 10.1024/0300-9831/a000528 30789808

[54] Asghari S Asghari-Jafarabadi M Somi MH Ghavami SM Rafraf M . Comparison of calorie-restricted diet and resveratrol supplementation on anthropometric indices, metabolic parameters, and serum sirtuin-1 levels in patients with nonalcoholic fatty liver disease: A randomized controlled clinical trial. J Am Coll Nutr (2018) 37(3):223–33. doi: 10.1080/07315724.2017.1392264 29313746

[55] Faghihzadeh F Adibi P Rafiei R Hekmatdoost A . Resveratrol supplementation improves inflammatory biomarkers in patients with nonalcoholic fatty liver disease. Nutr Res (2014) 34(10):837–43. doi: 10.1016/j.nutres.2014.09.005 25311610

[56] Faghihzadeh F Adibi P Hekmatdoost A . The effects of resveratrol supplementation on cardiovascular risk factors in patients with non-alcoholic fatty liver disease: A randomised, double-blind, placebo-controlled study. Br J Nutr (2015) 114(5):796–803. doi: 10.1017/S0007114515002433 26234526

[57] Kantartzis K Fritsche L Bombrich M Machann J Schick F Staiger H . Effects of resveratrol supplementation on liver fat content in overweight and insulin-resistant subjects: A randomized, double-blind, placebo-controlled clinical trial. Diabetes ObesMetab (2018) 20(7):1793–7. doi: 10.1111/dom.13268 29484808

[58] Namkhah Z Naeini F Mahdi Rezayat S Yaseri M Mansouri S . Javad hosseinzadeh-attar M.(2021) does naringenin supplementation improve lipid profile, severity of hepatic steatosis and probability of liver fibrosis in overweight/obese patients with NAFLD? A randomised, double-blind, placebo-controlled, clinical trial. Int J Clin Pract 75(11):e14852. doi: 10.1111/ijcp.14852 34516703

[59] Naeini F Namkhah Z Tutunchi H Rezayat SM Mansouri S Yaseri M . Effects of naringenin supplementation on cardiovascular risk factors in overweight/obese patients with nonalcoholic fatty liver disease: A pilot double-blind, placebo-controlled, randomized clinical trial. Eur J Gastroenterol Hepatol (2022) 34(3):345–53. doi: 10.1097/MEG.0000000000002323 34860705

[60] Sangsefidi ZS Hosseinzadeh M Ranjbar AM Akhondi-Meybodi M Fallahzadeh H Mozaffari-khosravi H . The effect of total anthocyanin-base standardized (Cornus mas l.) fruit extract on liver function, tumor necrosis factor α, malondealdehyde, and adiponectin in patients with non-alcoholic fatty liver: A study protocol for a double-blind randomized clinical trial. Nutr J (2019) 18(1):39. doi: 10.1186/s12937-019-0465-z 31324181 PMC6642510

[61] Yari Z Cheraghpour M Alavian SM Hedayati M Eini-Zinab H Hekmatdoost A . The efficacy of flaxseed and hesperidin on non-alcoholic fatty liver disease: An open-labeled randomized controlled trial. Eur J Clin Nutr (2021) 75(1):99–111. doi: 10.1038/s41430-020-0679-3 32647367

[62] Cheraghpour M Imani H Ommi S Alavian SM Karimi-Shahrbabak E Hedayati M . Hesperidin improves hepatic steatosis, hepatic enzymes, and metabolic and inflammatory parameters in patients with nonalcoholic fatty liver disease: A randomized, placebo-controlled, double-blind clinical trial. Phytother Res (2019) 33(8):2118–25. doi: 10.1002/ptr.6406 31264313

[63] Sakata R Nakamura T Torimura T Ueno T Sata M . Green tea with high-density catechins improves liver function and fat infiltration in non-alcoholic fatty liver disease (NAFLD) patients: A double-blind placebo-controlled study. Int J Mol Med (2013) 32(5):989–94. doi: 10.3892/ijmm.2013.1503 24065295

[64] Tabatabaee SM Alavian SM Ghalichi L Miryounesi SM Mousavizadeh K . Green tea in non-alcoholic fatty liver disease: A double blind randomized clinical trial. Hepat Mon (2017) 17(12):e14993. doi: 10.5812/hepatmon.14993

[65] Hussain M Rehman H-U Akhtar L . Therapeutic benefits of green tea extract on various parameters in non-alcoholic fatty liver disease patients. Pak J Med Sci (2017) 33(4):931–6. doi: 10.12669/pjms.334.12571 PMC5648967 29067068

[66] Federico A Dallio M Masarone M Gravina AG Di Sarno R Tuccillo C . Evaluation of the effect derived from silybin with vitamin d and vitamin e administration on clinical, metabolic, endothelial dysfunction, oxidative stress parameters, and serological worsening markers in nonalcoholic fatty liver disease patients. Oxid Med Cell Longev (2019) 2019:8742075. doi: 10.1155/2019/8742075 31737175 PMC6815609

[67] Loguercio C Andreone P Brisc C Brisc MC Bugianesi E Chiaramonte M . Silybin combined with phosphatidylcholine and vitamin e in patients with nonalcoholic fatty liver disease: A randomized controlled trial. Free Radic Biol Med (2012) 52(9):1658–65. doi: 10.1016/j.freeradbiomed.2012.02.008 22343419

[68] Wah Kheong C Nik Mustapha NR Mahadeva S . A randomized trial of silymarin for the treatment of nonalcoholic steatohepatitis. Clin Gastroenterol Hepatol (2017) 15(12):1940–1949.e8. doi: 10.1016/j.cgh.2017.04.016 28419855

[69] Hashemi S J Eskandar H Sardabi EH . A placebo-controlled trial of silymarin in patients with nonalcoholic fatty liver disease. Hepatitis Monthly (2009), 9(4): 265–70. doi: 10.1136/gut.2008.174516corr1

[70] Solhi H Ghahremani R Kazemifar AM HoseiniYazdi Z . Silymarin in treatment of non-alcoholic steatohepatitis: A randomized clinical trial. Caspian J Intern Med (2014) 5(1):9–12. 24490006 PMC3894463

[71] Anushiravani A Haddadi N Pourfarmanbar M Mohammadkarimi V . Treatment options for nonalcoholic fatty liver disease: A double-blinded randomized placebo-controlled trial. Eur J Gastroenterol Hepatol (2019) 31(5):613–7. doi: 10.1097/MEG.0000000000001369 30920975

[72] Navarro VJ Belle SH D’Amato M Adfhal N Brunt EM Fried MW . Silymarin in non-cirrhotics with non-alcoholic steatohepatitis: A randomized, double-blind, placebo controlled trial. PloS One (2019) 14(9):e0221683. doi: 10.1371/journal.pone.0221683 31536511 PMC6752871

[73] Zhong S Fan Y Yan Q Fan X Wu B Han Y . The therapeutic effect of silymarin in the treatment of nonalcoholic fatty disease: A meta-analysis (PRISMA) of randomized control trials. Med (Baltimore) (2017) 96(49):e9061. doi: 10.1097/MD.0000000000009061 PMC5728929 29245314

[74] Amanat S Eftekhari MH Fararouei M Bagheri Lankarani K Massoumi SJ . Genistein supplementation improves insulin resistance and inflammatory state in non-alcoholic fatty liver patients: A randomized, controlled trial. Clin Nutr (2018) 37(4):1210–5. doi: 10.1016/j.clnu.2017.05.028 28647291

[75] Mansour-Ghanaei F Pourmasoumi M Hadi A . Efficacy of curcumin/turmeric on liver enzymes in patients with non-alcoholic fatty liver disease: A systematic review of randomized controlled trials. Integr Med Res (2019) 8(1):57–61. doi: 10.1016/j.imr.2018.07.004 30949432 PMC6428926

[76] Poulsen MK Nellemann B Bibby BM Stødkilde-Jørgensen H Pedersen SB Grønbaek H . No effect of resveratrol on VLDL-TG kinetics and insulin sensitivity in obese men with nonalcoholic fatty liver disease. Diabetes ObesMetab (2018) 20(10):2504–9. doi: 10.1111/dom.13409 29885082

[77] Pierantonelli I Svegliati-Baroni G . Nonalcoholic fatty liver disease: Basic pathogenetic mechanisms in the progression from NAFLD to NASH. Transplantation (2019) 103(1):e1–e13. doi: 10.1097/TP.0000000000002480 30300287

[78] Cobbina E Akhlaghi F . Non-alcoholic fatty liver disease (NAFLD) - pathogenesis, classification, and effect on drug metabolizing enzymes and transporters. Drug Metab Rev (2017) 49(2):197–211. doi: 10.1080/03602532.2017.1293683 28303724 PMC5576152

[79] Castera L Friedrich-Rust M Loomba R . Noninvasive assessment of liver disease in patients with nonalcoholic fatty liver disease. Gastroenterology (2019) 156(5):1264–1281.e4. doi: 10.1053/j.gastro.2018.12.036 30660725 PMC7505052

[80] Manne V Handa P Kowdley KV . Pathophysiology of nonalcoholic fatty liver disease/nonalcoholic steatohepatitis. Clin Liver Dis (2018) 22(1):23–37. doi: 10.1016/j.cld.2017.08.007 29128059

[81] Gallego-Durán R Montero-Vallejo R Maya-Miles D Lucena A Martin F Ampuero J . Analysis of common pathways and markers from non-alcoholic fatty liver disease to immune-mediated diseases. Front Immunol (2021) 12:667354. doi: 10.3389/fimmu.2021.667354 34899679 PMC8652219

[82] Wang XJ Malhi H . Nonalcoholic fatty liver disease. Ann Intern Med (2018) 169(9):ITC65–80. doi: 10.7326/AITC201811060 30398639

[83] Polyzos SA Kountouras J Mantzoros CS . Obesity and nonalcoholic fatty liver disease: From pathophysiology to therapeutics. Metabolism (2019) 92:82–97. doi: 10.1016/j.metabol.2018.11.014 30502373

[84] Carr RM Oranu A Khungar V . Nonalcoholic fatty liver disease: Pathophysiology and management. Gastroenterol Clin North Am (2016) 45(4):639–52. doi: 10.1016/j.gtc.2016.07.003 PMC5127277 27837778

[85] Pafili K Roden M . Nonalcoholic fatty liver disease (NAFLD) from pathogenesis to treatment concepts in humans. Mol Metab (2021) 50:101122. doi: 10.1016/j.molmet.2020.101122 33220492 PMC8324683

[86] Eslam M Sanyal AJ George J . International consensus panel. MAFLD: A consensus-driven proposed nomenclature for metabolic associated fatty liver disease. Gastroenterology (2020) 158(7):1999–2014.e1. doi: 10.1053/j.gastro.2019.11.312 32044314

[87] Albillos A de Gottardi A Rescigno M . The gut-liver axis in liver disease: Pathophysiological basis for therapy. J Hepatol (2020) 72(3):558–77. doi: 10.1016/j.jhep.2019.10.003 31622696

[88] Safari Z Gérard P . The links between the gut microbiome and non-alcoholic fatty liver disease (NAFLD). Cell Mol Life Sci (2019) 76(8):1541–58. doi: 10.1007/s00018-019-03011-w PMC11105223 30683985

[89] Martín-Mateos R Albillos A . The role of the gut-liver axis in metabolic dysfunction-associated fatty liver disease. Front Immunol (2021) 12:660179. doi: 10.3389/fimmu.2021.660179 33936094 PMC8085382

[90] Svegliati-Baroni G Patrício B Lioci G Macedo MP Gastaldelli A . Gut-Pancreas-Liver axis as a target for treatment of NAFLD/NASH. Int J Mol Sci (2020) 21(16):5820. doi: 10.3390/ijms21165820 PMC7461212 32823659

[91] Aron-Wisnewsky J Warmbrunn MV Nieuwdorp M Clément K . Nonalcoholic fatty liver disease: Modulating gut microbiota to improve severity? Gastroenterology (2020) 158(7):1881–98. doi: 10.1053/j.gastro.2020.01.049 32044317

[92] Hundertmark J Krenkel O Tacke F . Adapted immune responses of myeloid-derived cells in fatty liver disease. Front Immunol (2018) 9:2418. doi: 10.3389/fimmu.2018.02418 30405618 PMC6200865

[93] Xu L Liu W Bai F Xu Y Liang X Ma C . Hepatic macrophage as a key player in fatty liver disease. Front Immunol (2021) 12:708978. doi: 10.3389/fimmu.2021.708978 34956171 PMC8696173

[94] Rinella ME . Nonalcoholic fatty liver disease: A systematic review. JAMA (2015) 313(22):2263–73. doi: 10.1001/jama.2015.5370 26057287

[95] Ganesan K Xu B . Polyphenol-rich lentils and their health promoting effects. Int J Mol Sci (2017) 18(11):2390. doi: 10.3390/ijms18112390 PMC5713359 29125587

[96] Wan MLY Co VA El-Nezami H . Dietary polyphenol impact on gut health and microbiota. Crit Rev Food Sci Nutr (2021) 61(4):690–711. doi: 10.1080/10408398.2020.1744512 32208932

[97] Guo Y Sun Q Wu FG Dai Y Chen X . Polyphenol-containing nanoparticles: Synthesis, properties, and therapeutic delivery. Adv Mater (2021) 33(22):e2007356. doi: 10.1002/adma.202007356 33876449

[98] Myburgh KH . Polyphenol supplementation: Benefits for exercise performance or oxidative stress? Sports Med (2014) 44 Suppl 1(Suppl 1):S57–70. doi: 10.1007/s40279-014-0151-4 PMC4008802 24791917

[99] Hussain T Tan B Yin Y Blachier F Tossou MC Rahu N . Oxidative stress and inflammation: What polyphenols can do for us? Oxid Med Cell Longev (2016) 2016:7432797. doi: 10.1155/2016/7432797 27738491 PMC5055983

[100] Babu PV Liu D Gilbert ER . Recent advances in understanding the anti-diabetic actions of dietary flavonoids. J Nutr Biochem (2013) 24(11):1777–89. doi: 10.1016/j.jnutbio.2013.06.003 PMC3821977 24029069

[101] Wang S Moustaid-Moussa N Chen L Mo H Shastri A Su R . Novel insights of dietary polyphenols and obesity. J Nutr Biochem (2014) 25(1):1–18. doi: 10.1016/j.jnutbio.2013.09.001 24314860 PMC3926750

[102] Rafiei H Omidian K Bandy B . Dietary polyphenols protect against oleic acid-induced steatosis in an in vitro model of nafld by modulating lipid metabolism and improving mitochondrial function. Nutrients (2019) 11(3):541. doi: 10.3390/nu11030541 PMC6471211 30832407

[103] Rafiei H Omidian K Bandy B . Comparison of dietary polyphenols for protection against molecular mechanisms underlying nonalcoholic fatty liver disease in a cell model of steatosis. Mol Nutr Food Res (2017) 61(9):1–12. doi: 10.1002/mnfr.201600781 28317281

[104] Bhattacharjee S Dashwood RH . Epigenetic regulation of NRF2/KEAP1 by phytochemicals. Antioxid (Basel) (2020) 9(9):865. doi: 10.3390/antiox9090865 PMC7555619 32938017

[105] Grilc NK Sova M Kristl J . Drug delivery strategies for curcumin and other natural nrf2 modulators of oxidative stress-related diseases. Pharmaceutics (2021) 13(12):2137. doi: 10.3390/pharmaceutics13122137 34959418 PMC8708625

[106] Li HY Gan RY Shang A Mao QQ Sun QC Wu DT . Plant-based foods and their bioactive compounds on fatty liver disease: Effects, mechanisms, and clinical application. Oxid Med Cell Longev (2021) 2021:6621644. doi: 10.1155/2021/6621644 33728021 PMC7939748

[107] Nelson KM Dahlin JL Bisson J Graham J Pauli GF Walters MA . The essential medicinal chemistry of curcumin. J Med Chem (2017) 60(5):1620–37. doi: 10.1021/acs.jmedchem.6b00975 PMC5346970 28074653

[108] Jabczyk M Nowak J Hudzik B Zubelewicz-Szkodzińska B . Curcumin in metabolic health and disease. Nutrients (2021) 13(12):4440. doi: 10.3390/nu13124440 34959992 PMC8706619

[109] Różański G Kujawski S JL N Zalewski P Słomko J . Curcumin and biochemical parameters in metabolic-associated fatty liver disease (MAFLD)-a review. Nutrients (2021) 13(8):2654. doi: 10.3390/nu13082654 34444811 PMC8401796

[110] Zabihi NA Pirro M Johnston TP Sahebkar A . Is there a role for curcumin supplementation in the treatment of non-alcoholic fatty liver disease? the data suggest yes. Curr Pharm Des (2017) 23(7):969–82. doi: 10.2174/1381612822666161010115235 27748192

[111] Xu G Huang K Zhou J . Hepatic AMP kinase as a potential target for treating nonalcoholic fatty liver disease: Evidence from studies of natural products. Curr Med Chem (2018) 25(8):889–907. doi: 10.2174/0929867324666170404142450 28393690

[112] Liu Y Cheng F Luo Y Zhan Z Hu P Ren H . PEGylated curcumin derivative attenuates hepatic steatosis *via* CREB/PPAR-γ/CD36 pathway. BioMed Res Int (2017) 2017:8234507. doi: 10.1155/2017/8234507 28770225 PMC5523402

[113] Zhao M Chen S Ji X Shen X You J Liang X . Current innovations in nutraceuticals and functional foods for intervention of non-alcoholic fatty liver disease. Pharmacol Res (2021) 166:105517. doi: 10.1016/j.phrs.2021.105517 33636349

[114] Ravindranath V Chandrasekhar N . Absorption and tissue distribution of curcumin in rats. Toxicology (1980) 16(3):259–66. doi: 10.1016/0300-483X(80)90122-5 7423534

[115] Wahlstrom B Blennow G . A study on the fate of curcumin in the rat. Acta Pharmacol Toxicol (1978) 43(2):86–92. doi: 10.1111/j.1600-0773.1978.tb02240.x 696348

[116] Sugimoto K Hanai H Tozawa K Aoshi T Uchijima M Nagata T . Curcumin prevents and ameliorates trinitrobenzene sulfonic acid-induced colitis in mice. Gastroenterology (2002) 123(6):1912–22. doi: 10.1053/gast.2002.37050 12454848

[117] Chainani-Wu N . Safety and anti-inflammatory activity of curcumin: a component of tumeric (Curcuma longa). J Altern Complement Med (2003) 9(1):161–8. doi: 10.1089/107555303321223035 12676044

[118] Cheng AL Hsu CH Lin JK Hsu MM Ho YF Shen TS . Phase I clinical trial of curcumin, A chemopreventive agent, in patients with high-risk or pre-malignant lesions. Anticancer Res (2001) 21(4B):2895–900. 11712783

[119] Hosseini H Teimouri M Shabani M Koushki M BabaeiKhorzoughi R Namvarjah F . Resveratrol alleviates non-alcoholic fatty liver disease through epigenetic modification of the Nrf2 signaling pathway. Int J Biochem Cell Biol (2020) 119:105667. doi: 10.1016/j.biocel.2019.105667 31838177

[120] Huang Y Lang H Chen K Zhang Y Gao Y Ran L . Resveratrol protects against nonalcoholic fatty liver disease by improving lipid metabolism and redox homeostasis via the PPARα pathway. Appl Physiol Nutr Metab (2020) 45(3):227–39. doi: 10.1139/apnm-2019-0057 31173696

[121] Tejada S Capó X Mascaró CM Monserrat-Mesquida M Quetglas-Llabrés MM Pons A . Hepatoprotective effects of resveratrol in non-alcoholic fatty live disease. Curr Pharm Des (2021) 27(22):2558–70. doi: 10.2174/1381612826666200417165801 32303170

[122] Shang J Chen LL Xiao FX Sun H Ding HC Xiao H . Resveratrol improves non-alcoholic fatty liver disease by activating AMP-activated protein kinase. Acta Pharmacol Sin (2008) 29(6):698–706. doi: 10.1111/j.1745-7254.2008.00807.x 18501116

[123] Elgebaly A Radwan IA AboElnas MM Ibrahim HH Eltoomy MF Atta AA . Resveratrol supplementation in patients with non-alcoholic fatty liver disease: Systematic review and meta-analysis. J Gastrointestin Liver Dis (2017) 26:59–67. doi: 10.15403/jgld.2014.1121.261.ely 28338115

[124] Zhang X Zhang Y Gao W Guo Z Wang K Liu S . Naringin improves lipid metabolism in a tissue-engineered liver model of NAFLD and the underlying mechanisms. Life Sci (2021) 277:119487. doi: 10.1016/j.lfs.2021.119487 33862107

[125] Zhang J Zhao L Cheng Q Ji B Yang M Sanidad KZ . Structurally different flavonoid subclasses attenuate high-fat and high-fructose diet induced metabolic syndrome in rats. J Agric Food Chem (2018) 66(46):12412–20. doi: 10.1021/acs.jafc.8b03574 30360615

[126] Rodríguez V Plavnik L Tolosa de Talamoni N . Naringin attenuates liver damage in streptozotocin-induced diabetic rats. BioMed Pharmacother (2018) 105:95–102. doi: 10.1016/j.biopha.2018.05.120 29852394

[127] Mu H Zhou Q Yang R Zeng J Li X Zhang R Dong J . Naringin attenuates high fat diet induced non-alcoholic fatty liver disease and gut bacterial dysbiosis in mice. Front Microbiol (2020) 11:585066. doi: 10.3389/fmicb.2020.585066 33281780 PMC7691324

[128] Shin JH Jung JH . Non-alcoholic fatty liver disease and flavonoids: Current perspectives. Clin Res Hepatol Gastroenterol (2017) 41(1):17–24. doi: 10.1016/j.clinre.2016.07.001 27545758

[129] Kalt W Cassidy A Howard LR Krikorian R Stull AJ Tremblay F . Recent research on the health benefits of blueberries and their anthocyanins. Adv Nutr (2020) 11(2):224–36. doi: 10.1093/advances/nmz065 PMC7442370 31329250

[130] Lee YM Yoon Y Yoon H Park HM Song S Yeum KJ . Dietary anthocyanins against obesity and inflammation. Nutrients (2017) 9(10):1089. doi: 10.3390/nu9101089 PMC5691706 28974032

[131] Cladis DP Weaver CM Ferruzzi MG . (Poly)phenol toxicity in vivo following oral administration: A targeted narrative review of (poly)phenols from green tea, grape, and anthocyanin-rich extracts. Phytother Res (2022) 36(1):323–35. doi: 10.1002/ptr.7323 34725890

[132] Fang J . Bioavailability of anthocyanins. Drug Metab Rev (2014) 46(4):508–20. doi: 10.3109/03602532.2014.978080 25347327

[133] Sharma S Katoch V Kumar S Chatterjee S . Functional relationship of vegetable colors and bioactive compounds: Implications in human health. J Nutr Biochem (2021) 92:108615. doi: 10.1016/j.jnutbio.2021.108615 33705954

[134] Mehmood A Zhao L Wang Y Pan F Hao S Zhang H . Dietary anthocyanins as potential natural modulators for the prevention and treatment of non-alcoholic fatty liver disease: A comprehensive review. Food Res Int (2021) 142:110180. doi: 10.1016/j.foodres.2021.110180 33773656

[135] Valenti L Riso P Mazzocchi A Porrini M Fargion S . Agostoni C. dietary anthocyanins as nutritional therapy for nonalcoholic fatty liver disease. Oxid Med Cell Longev (2013), 145421. doi: 10.1155/2013/145421 24282628 PMC3824564

[136] Xiong H Wang J Ran Q Lou G Peng C Gan Q . Hesperidin: A therapeutic agent for obesity. Drug Des Devel Ther (2019) 13:3855–66. doi: 10.2147/DDDT.S227499 PMC6859214 32009777

[137] Li C Schluesener H . Health-promoting effects of the citrus flavanone hesperidin. Crit Rev Food Sci Nutr (2017) 57(3):613–31. doi: 10.1080/10408398.2014.906382 25675136

[138] Dokumacioglu E Iskender H Musmul A . Effect of hesperidin treatment on α-Klotho/FGF-23 pathway in rats with experimentally-induced diabetes. BioMed Pharmacother (2019) 109:1206–10. doi: 10.1016/j.biopha.2018.10.192 30551370

[139] Wang SW Sheng H Bai YF Weng YY Fan XY Lou LJ . Neohesperidin enhances PGC-1α-mediated mitochondrial biogenesis and alleviates hepatic steatosis in high fat diet fed mice. Nutr Diabetes (2020) 10(1):27. doi: 10.1038/s41387-020-00130-3 32759940 PMC7406515

[140] Sukkasem N Chatuphonprasert W Jarukamjorn K . Hesperidin, a novel candidate for the successful treatment of high fat diet plus ethanol-induced fatty liver disease in mice. J Physiol Pharmacol (2021) 72(2):217–24. doi: 10.26402/jpp.2021.2.07 34374658

[141] Xie Q Gao S Lei M . Hesperidin suppresses ERS-induced inflammation in the pathogenesis of non-alcoholic fatty liver disease. Aging (Albany NY) (2022) 14(3):1265–79. doi: 10.18632/aging.203817 PMC8876922 35143415

[142] Hodges JK Sasaki GY . Anti-inflammatory activities of green tea catechins along the gut-liver axis in nonalcoholic fatty liver disease: Lessons learned from preclinical and human studies. J Nutr Biochem (2020) 85:108478. doi: 10.1016/j.jnutbio.2020.108478 32801031

[143] Musial C Kuban-Jankowska A . Gorska-ponikowska M.(2020) beneficial properties of green tea catechins. Int J Mol Sci 21(5):1744. doi: 10.3390/ijms21051744 PMC7084675 32143309

[144] Bansal S Vyas S Bhattacharya S Sharma M . Catechin prodrugs and analogs: A new array of chemical entities with improved pharmacological and pharmacokinetic properties. Nat Prod Rep (2013) 30(11):1438–54. doi: 10.1039/c3np70038k 24056761

[145] Masterjohn C Bruno RS . Therapeutic potential of green tea in nonalcoholic fatty liver disease. Nutr Rev (2012) 70(1):41–56. doi: 10.1111/j.1753-4887.2011.00440.x 22221215

[146] Tang G Xu Y Zhang C Wang N Li H Feng Y . Green tea and epigallocatechin gallate (EGCG) for the management of nonalcoholic fatty liver diseases (NAFLD): Insights into the role of oxidative stress and antioxidant mechanism. Antioxid (Basel) (2021) 10(7):1076. doi: 10.3390/antiox10071076 PMC8301033 34356308

[147] Xin X Cheng C Bei-Yu C Hong-Shan L Hua-Jie T Xin W . Caffeine and egcg alleviate high-trans fatty acid and high-carbohydrate diet-induced nash in mice: Commonality and specificity. Front Nutr (2021) 8:784354. doi: 10.3389/fnut.2021.784354 34881283 PMC8647766

[148] Mahmoodi M Hosseini R Kazemi A Ofori-Asenso R Mazidi M Mazloomi SM . Effects of green tea or green tea catechin on liver enzymes in healthy individuals and people with nonalcoholic fatty liver disease: A systematic review and meta-analysis of randomized clinical trials. Phytother Res (2020) 34(7):1587–98. doi: 10.1002/ptr.6637 32067271

[149] Camini FC Costa DC . Silymarin: Not just another antioxidant. J Basic Clin Physiol Pharmacol (2020) 31(4):1–12. doi: 10.1515/jbcpp-2019-0206 32134732

[150] Federico A Dallio M . Silymarin/Silybin and chronic liver disease: A marriage of many years. Molecules (2017) 22(2):191. doi: 10.3390/molecules22020191 PMC6155865 28125040

[151] Sahin E Bagci R BekturAykanat NE Kacar S Sahinturk V . Silymarin attenuated nonalcoholic fatty liver disease through the regulation of endoplasmic reticulum stress proteins GRP78 and XBP-1 in mice. J Food Biochem (2020) 44(6):e13194. doi: 10.1111/jfbc.13194 32189355

[152] Abenavoli L Izzo AA Milić N Cicala C Santini A Capasso R . Milk thistle (Silybum marianum): A concise overview on its chemistry, pharmacological, and nutraceutical uses in liver diseases. Phytother Res (2018) 32(11):2202–13. doi: 10.1002/ptr.6171 30080294

[153] Salvoza N Giraudi PJ Tiribelli C Rosso N . Natural compounds for counteracting nonalcoholic fatty liver disease (nafld): Advantages and limitations of the suggested candidates. Int J Mol Sci (2022) 23(5):2764. doi: 10.3390/ijms23052764 35269912 PMC8911502

[154] Jaiswal N Akhtar J Singh SP Badruddeen Ahsan F . An overview on genistein and its various formulations. Drug Res (Stuttg) (2019) 69(6):305–13. doi: 10.1055/a-0797-3657 30517965

[155] Yu L Rios E Castro L Liu J Yan Y Dixon D . Genistein: Dual role in women’s health. Nutrients (2021) 13(9):3048. doi: 10.3390/nu13093048 34578926 PMC8472782

[156] Bhat SS Prasad SK Shivamallu C Prasad KS Syed A Reddy P . Genistein: A potent anti-breast cancer agent. Curr Issues Mol Biol (2021) 43(3):1502–17. doi: 10.3390/cimb43030106 PMC8929066 34698063

[157] Wei TT Chandy M Nishiga M Zhang A Kumar KK Thomas D . Cannabinoid receptor 1 antagonist genistein attenuates marijuana-induced vascular inflammation. Cell (2022) 185(10):1676–1693.e23. doi: 10.1016/j.cell.2022.04.005 35489334 PMC9400797

[158] Sharifi-Rad J Quispe C Imran M Rauf A Nadeem M Gondal TA . Genistein: An integrative overview of its mode of action, pharmacological properties, and health benefits. Oxid Med Cell Longev (2021) 2021:3268136. doi: 10.1155/2021/3268136 34336089 PMC8315847

[159] Mas-Bargues C Borrás C Viña J . Genistein, a tool for geroscience. Mech Ageing Dev (2022) 204:111665. doi: 10.1016/j.mad.2022.111665 35307412

[160] Sureda A Sanches Silva A Sánchez-Machado DI López-Cervantes J Daglia M Nabavi SF . Hypotensive effects of genistein: From chemistry to medicine. Chem Biol Interact (2017) 268:37–46. doi: 10.1016/j.cbi.2017.02.012 28242380

[161] Ji G Yang Q Hao J Guo L Chen X Hu J . Anti-inflammatory effect of genistein on non-alcoholic steatohepatitis rats induced by high fat diet and its potential mechanisms. Int Immunopharmacol (2011) 11(6):762–8. doi: 10.1016/j.intimp.2011.01.036 21320636

