# Supplementary material for: Efficacy and safety of dietary polyphenol supplementation in the treatment of non-alcoholic fatty liver disease: A systematic review and meta-analysis
Source: Front Immunol. 2022 Sep 9;13:949746. doi: 10.3389/fimmu.2022.949746 (PMC9500378; doi:10.3389/fimmu.2022.949746)
Supplement: Supplementary file 1 [file Table_1.docx]

**Table S1.** Search Strategies for Pubmed and Embase

| **PubMed** | ((Polyphenols OR Polyphenol OR Provinols) OR (Curcumin OR Curcumas OR Tumeric OR Tumerics OR Turmeric OR Turmerics OR Curcuma zedoaria OR Curcuma zedoarias OR zedoaria, Curcuma OR Zedoary zedoaria OR Zedoary zedoarias OR zedoaria, Zedoary OR Curcuma longa OR Curcuma longas OR longa, Curcuma OR Curcuma Longa) OR (resveratrol OR resveratrols OR 3,4',5-stilbenetriol OR 3,5,4'-trihydroxystilbene OR trans-resveratrol-3-O-sulfate OR SRT 501 OR SRT501 OR SRT-501 OR trans-resveratrol OR cis-resveratrol OR resveratrol-3-sulfate) OR (Naringenin OR naringenin-7-sulfate) OR (Anthocyanin OR Anthocyanins OR Leucoanthocyanidins OR Anthocyanidin) OR (Hesperidin OR Hesperetin 7-Rhamnoglucoside OR 7-Rhamnoglucoside, Hesperetin OR Hesperetin 7 Rhamnoglucoside OR Hesperetin-7-Rutinoside OR Hesperetin 7 Rutinoside OR Hesperidin 2S) OR (Catechin OR Cianidanol OR Catechinic Acid OR Catechuic Acid OR Catergen OR Zyma OR Epicatechin OR KB-53 OR KB 53 OR KB53 OR Z 7300 OR Cyanidanol-3 OR Cyanidanol 3) OR (Silymarin OR Silimarin OR Karsil OR Legalon OR Carsil))  AND  (Non-alcoholic Fatty Liver Disease OR Non alcoholic Fatty Liver Disease OR NAFLD OR Nonalcoholic Fatty Liver Disease OR Fatty Liver, Nonalcoholic OR Fatty Livers, Nonalcoholic OR Liver, Nonalcoholic Fatty OR Livers, Nonalcoholic Fatty OR Nonalcoholic Fatty Liver OR Nonalcoholic Fatty Livers OR Nonalcoholic Steatohepatitis OR Nonalcoholic Steatohepatitides OR Steatohepatitides, Nonalcoholic)  AND  (random* controlled trial [pt] OR controlled clinical trial* [pt] OR randomized [tiab] OR placebo [tiab] OR drug therapy [sh] OR random* [tiab] OR trial* [tiab] OR group* [tiab])  NOT  (animals [mh] NOT humans [mh]) |
| --- | --- |
| **EMBASE** | 1 Polyphenols/  2 Polyphenol/  3 Provinols/  4 1-3/or  5 Curcumin/  6 Curcumas/  7 Tumeric/  8 Tumerics/  9 Turmeric/  10 Turmerics/  11 Zedoary zedoaria/  12 Zedoary zedoarias Curcuma longa/  13 Curcuma zedoaria/  14 Curcuma zedoarias/  15 Curcuma longas/  16 Curcuma Longa/  17 5-16/or  18 resveratrol/  19 resveratrols/  20 trans-resveratrol-3-O-sulfate/  21 trans-resveratrol/  22 cis-resveratrol/  23 resveratrol-3-sulfate/  24 18-23/or  25 Naringenin/  26 naringenin-7-sulfate/  27 25 or 26  28 Anthocyanin/  29 Anthocyanins/  30 Leucoanthocyanidins/  31 Anthocyanidin/  32 28-31/or  33 Hesperidin/  34 Hesperetin 7-Rhamnoglucoside/  35 7-Rhamnoglucoside, Hesperetin/  36 Hesperetin 7 Rhamnoglucoside/  37 Hesperetin-7-Rutinoside/  38 Hesperetin 7 Rutinoside/  39 Hesperidin 2S/  40 33-39/or  41 Catechin/  42 Cianidanol/  43 Catechinic Acid/  44 Catechuic Acid/  45 Catergen/  46 Zyma/  47 Epicatechin/  48 KB-53/  49 KB 53/  50 KB53/  51 Z 7300/  52 Cyanidanol-3/  53 Cyanidanol 3/  54 41-53/or  55 Silymarin/  56 Silimarin/  57 Karsil/  58 Legalon/  59 Carsil/  60 55-59/or  61 4 or 17 or 24 or 27 or 32 or 40 or 54 or 60  62 Non-alcoholic Fatty Liver Disease/  63 Non alcoholic Fatty Liver Disease/  64 NAFLD/  65 Nonalcoholic Fatty Liver Disease/  66 Fatty Liver, Nonalcoholic/  67 Fatty Livers, Nonalcoholic/  68 Liver, Nonalcoholic Fatty/  69 Livers, Nonalcoholic Fatty/  70 Nonalcoholic Fatty Liver/  71 Nonalcoholic Fatty Livers/  72 Nonalcoholic Steatohepatitis/  73 Nonalcoholic Steatohepatitides/  74 Steatohepatitides, Nonalcoholic/  75 62-74/or  76 crossover procedure/  77 double blind procedure/  78 single blind procedure/  79 triple blind procedure/  80 randomized controlled trial/  81 76-80/or  82 61 and 75 and 81 |
